# Srebf2 mediates successful optic nerve axon regeneration via the mevalonate synthesis pathway

**DOI:** 10.1186/s13024-025-00807-2

**Published:** 2025-03-05

**Authors:** Mengming Hu, Matthew B. Veldman

**Affiliations:** 1 Department of Cell Biology, Neurobiology, and Anatomy, Medical College of Wisconsin, 8701 Watertown Plank Road, Milwaukee, WI 53226 USA; 2 Department of Ophthalmology and Visual Science, Medical College of Wisconsin, Milwaukee, USA

**Keywords:** Axon, Retinal ganglion cell, Cholesterol, Mevalonate, Optic nerve, Regeneration, srebf2, Zebrafish

## Abstract

**Background:**

Axon regeneration within the mammalian central nervous system is extremely limited. In optic neuropathy conditions like glaucoma, the inability of retinal ganglion cell (RGC) axons to regenerate is a major impediment to functional recovery. In contrast, adult teleost fish such as zebrafish can fully regenerate RGC axons enabling visual recovery from optic nerve (ON) injury making it an ideal model to probe the mechanisms of successful axon regeneration.

**Methods:**

Laser Capture Microdissection followed by RNA-sequencing (LCM-seq) was used to identify genes and pathways differentially expressed in RGCs during ON regeneration. We validate these findings by in situ hybridization and qRT-PCR. Using loss- and gain-of-function experiments we demonstrate the necessity of *srebf2* for efficient ON regeneration and recovery of visual function. Finally, we use LCM-seq coupled with experimental manipulations to identify downstream *srebf2* target genes and test the role of *hmgcra/b* and mevalonate in this process. Statistical analysis was performed using Student’s t-test, two-way ANOVA, or repeated measures with appropriate post-hoc tests where applicable.

**Results:**

LCM-seq comparison of uninjured versus 3-day post ON injury RGCs identified significant upregulation of the cholesterol synthesis pathway during axon regeneration. The master regulator of this pathway, the transcription factor *srebf2*, is upregulated throughout the regeneration phase. Chemical inhibition or morpholino-based gene knockdown of *srebf2* decreased axon regeneration into the ON and optic tectum and delayed recovery of visual behavior over the course of normal optic nerve regeneration without causing a significant loss of RGCs. Constitutively active *srebf2* can fully rescue axon regeneration and visual behavior losses caused by inhibition of endogenous *srebf2* but does not accelerate regeneration compared to the control group. LCM-seq confirms the expected regulation of predicted *srebf2* target genes after loss- or gain-of-function in vivo. Downstream of *srebf2*, *hmgcra/b* knockdown or simvastatin treatment delayed axon regeneration and this effect was rescued by supplemental mevalonate. Mevalonate treatment alone was sufficient to accelerate ON regeneration.

**Conclusions:**

These results demonstrate that *srebf2* and the downstream mevalonate synthesis pathway plays an important role in regulating efficient axon regeneration in the zebrafish visual system. Involvement of this pathway should be closely examined in failed mammalian ON regeneration.

**Supplementary Information:**

The online version contains supplementary material available at 10.1186/s13024-025-00807-2.

## Background

The inability of the adult mammalian central nervous system (CNS) to regenerate lost connections prevents significant functional recovery following injury or disease. In the visual system, ON injury caused by optic neuropathies, including glaucoma, leads to permanent vision loss with no available treatment to stimulate recovery [1]. To re-establish vision following ON injury, the axon must regrow, successfully navigate to its previous brain targets, and synaptically integrate in a stereotypic manner. This process is difficult to study in mammalian models since their CNS cannot naturally regenerate after injury. Alternatively, frog and fish models, including the commonly used zebrafish, can regenerate CNS axons, leading to the return of sensory functions, including vision [2]. This provides a unique opportunity to identify the cellular and molecular processes necessary for successful CNS regeneration.

Transcriptomic analysis of ON regeneration in pro-regenerative models is a logical starting point for the identification of the genes and pathways driving this process. LCM-microarray, whole retina RNA-seq, and FACS-seq have been previously performed at single or multiple time points during the ON regeneration process in zebrafish [3–8]. These studies have identified tens to hundreds of differentially expressed genes (DEGs) but have been limited by the genomic coverage of microarray chips or the abundance of RGCs in total retina samples (< 5% of the total cell population). The most recent FACS-seq study of purified RGCs is limited to early injury response time points, < 24 h post injury [8]. To explore the DEGs and pathways involved in rapid axon extension, we present here LCM-seq data of the ganglion cell layer (GCL) from 3 days post-injury (dpi) and uninjured retinas. This time point corresponds to the axon extension phase through the optic nerve up to the optic chiasm, and the LCM-seq technique allows us to assay transcriptome-wide changes at high read depth in an enriched population of RGCs with minimal tissue processing [9].

Gene Ontology and pathway analysis of DEGs at 3 dpi suggest that the cholesterol (chol) synthesis pathway is highly upregulated. The chol pathway consists of a multistep enzymatic synthesis of products including cholesterol, isoprenoids, heme A, dolichol, and ubiquinone [10]. These molecules are likely to have important roles in neural development, degeneration, and axon regeneration [11–13]. However, there are conflicting reports on the function of this pathway and its products on axon development and regeneration in the nervous system of mammals. Inhibition of the chol pathway during development results in early embryonic lethality in vivo [14] and reduces axon growth rate in primary cell culture in vitro [15]. Surprisingly, there are reports that statin drugs, which inhibit the first step in the chol pathway, increase axon regeneration in adults [16–19]. And this effect seems to be related to the balance of cholesterol at inhibitory lipid raft zones and protein prenylation of pro-regenerative proteins [18–20]. In the pro-regenerative adult goldfish ON regeneration model, cholesterol synthesis is necessary for axon growth from preconditioned retinal explants but not for axon regeneration in vivo [21]. The involvement of the alternative products of the chol pathway has not been thoroughly examined in this model, nor have the upstream metabolites. Given the conflicting data in mammalian models and incomplete information in pro-regenerative models, our goal here is to test the chol pathway’s role in CNS regeneration using the experimentally amenable zebrafish ON injury model and begin identifying the relevant metabolic products.

To determine the necessity of this pathway in successful CNS regeneration, we first test the role of *srebf2*, the master transcriptional regulator of the chol synthesis pathway [22]. Using in vivo axonal regeneration and visual recovery assays, we demonstrate that Srebf2 is necessary for efficient axon regeneration in RGCs. We then examine the importance of the mevalonate synthesis pathway, the first steps in the chol pathway, downstream of the *srebf2*-mediated effects using loss-of-function and rescue experiments. Our findings illustrate the importance of *srebf2* in the upregulation of the chol pathway for successful axon regeneration in adult zebrafish.

## Methods

### Zebrafish husbandry and surgery

Zebrafish were obtained from our breeding colony and maintained at 28 °C with a 14/10-h light/dark cycle. For all experiments performed on adult zebrafish, in each cohort, zebrafish at the same age ranging from 6 to 12 months old were used. For optic nerve crush, the fish were anesthetized by immersion in tricaine (0.033%, E10521, Sigma-Aldrich, USA). The right optic nerve was exposed by gently pulling the eye out of the orbit and cutting the connective tissue and ocular muscles around the dorsal half of the orbit. The nerve was then crushed with a number 5 forceps until the myelin was separated and the optic nerve sheath appeared clear at the crushed site, indicating all the fibers had been severed. For intraocular injection, fish were anesthetized with tricaine. A small incision is made in the posterior cornea adjacent to the lens with a double-edged sapphire scalpel (World Precision Instruments, USA). Then 0.5 μl of vehicle (PBS) or mevalonolactone (1 mM in PBS, M4667, Sigma-Aldrich, USA) was delivered using a blunt-end 33-gauge Hamilton (USA) syringe. Mevalonolactone is purchased as a powder and when placed in solution becomes mevalonate. All protocols were in accordance with the National Institutes of Health guidelines and the Association for Research in Vision and Ophthalmology (ARVO) statement for the use of animals in ophthalmic and vision research and were approved by the Institutional Animal Care and Use Committee at the Medical College of Wisconsin.

### Generation of nuclear Srebf2 heat shock-inducible transgenic zebrafish

Transgenic zebrafish were generated using the Tol2-based Multisite Gateway system [23]. Briefly, zebrafish nuclear *srebf2* (The first 460aa of the N-terminus comprising the bHLH domain but excluding the transmembrane domain and C-terminus) cDNA was cloned into the pDONR221 vector using primers nSrebf2 B1F (GGGGACAAGTTTGTACAAAAAAGCAGGCT gccaccATGGACGCCTCGGAGTTTATG) and nSrebf2 B2R (GGGGACCACTTTGTACAAGAAAGCTGGGTa cagcagcagacgagagc). The resulting plasmid, pME-*nSrebf2*, was combined with p5E-*hsp70l*, p3E-*2AmCherry* and pDestTol2CG2 (EGFP driven by the *cmlc2* promoter) in an LR reaction resulting in *Tg(hsp70l: nSrebf2-2AmCherry*,* cmlc2:EGFP)*. To generate transgenic fish, 50 pg of *Tg(hsp70l: nSrebf2-2AmCherry*,* cmlc2:EGFP)* with 25 pg of Tol2 transposase mRNA was injected into one-cell stage zebrafish embryos. The larvae with GFP fluorescence in the heart were selected and raised to adulthood. Then F_0_*Tg(hsp70l: nSrebf2-2AmCherry*,* cmlc2:EGFP)* were outcrossed with wildtype or *Tg(3.6fgap43:GFP)*^*SA1*^ [24] zebrafish for experiments.

### Morpholino, drug, and heat shock treatments

To facilitate Morpholino (MO) treatment, a partial ON transection, cutting about 50% of the width through the nerve sheath, was made at the crushed site immediately (within less than 1 minute) after injury, and a small piece of gel foam soaked in MO was placed on the distal ON stump. This treatment method partially preserves the proximal to distal connection of the optic nerve allowing regenerating axons to find their way back to the brain. We avoided performing complete ON transection since the nerve stump doesn’t always reconnect making quantification of axon regrowth difficult. The eye was then gently replaced in orbit, and the fish were placed in their home tank, allowing the MO to be retrogradely transported backward to the RGCs. After 3 hours, the fish were anesthetized, and the gel foam was taken out. The left nerve was left intact, and the left retina was used as an uninjured control. MO used for experiments include, (*srebf2*-MO1 5’-TGTCCTCGGATGCTCTTTCAAAAAG-3’, *srebf2*-MO2 5’-TGTGGTCAGACTCACCTGTGTGATT-3’, *hmgcra*-MO 5’ - ATTCGGAAAAGTCTCGTCAGCATGG − 3’, *hmgcrb*-MO 5’ - GCCTGAAGAGACGCGACAGCATCAT − 3’, and Standard control-MO 5’- CCTCTTACCTCAGTTACAATTTATA-3’) purchased from the Gene Tools (USA). For drug treatments, DMSO dissolved stock solution of fatostatin (stock: 20mM, working: 2 μM, F8932, MilliporeSigma, USA), or simvastatin (stock: 100 μM, working: 5 nM, S6196, MilliporeSigma, USA) were diluted in 400 ml of fish water. Fish water with the drug was changed every other day. At 7 days post-crush, fish were euthanized by an overdose of tricaine, and the eyes and brain were removed for further examination. For the heat shock procedure, heat treatment consisted of raising the water temperature from 28.5 °C to 38 °C over 20 min, then maintaining it at 38 °C for 30 min, and finally decreasing it to 28.5 °C over the next 15 min in the water bath [25].

### Laser capture microdissection and RNA extraction for RNA sequencing

The retina preparation, LCM, and RNA extraction were based on our established protocol [9]. Briefly, fresh frozen eyes with the lens removed were cryosectioned at 14 μm, mounted on slides covered with polyethylene-naphthalate (PEN)-membrane (415190-9081-000, Zeiss, Germany) and left to dry overnight at room temperature. Cresyl violet (C5042, Sigma) staining was performed. The GCL is visualized and microdissected for RNA extraction using the Ziess PALM LCM system. Small portions of the retinal fiber layer and inner plexiform layer adjacent to the cresyl violet positive GCL were included to maximize RNA yield. The entire GCL was collected from 12 to 15 sections that were equally distributed across the central 50% of the eye diameter, approximately 0.45 to 0.6 mm^2^ per fish. The NucleoSpin^®^ RNA XS kit (740902.50, Takara Bio) was used for RNA extraction. Total RNA collected from each sample ranged from ∼ 250 pg to 3 ng with uninjured samples on the low end and 3 dpi samples on the higher side. The quality of extracted RNA was assayed by 4200 TapeStation System (Agilent) using high sensitivity RNA screen tape. RNA samples with RIN score ≥ 8.0 were used for RNA sequencing.

### RNA sequencing analysis

Total RNA from LCM was collected to analyze the transcriptome. mRNA was amplified using the SMART-Seq v4 Ultra Low Input RNA Kit for Sequencing (Takara) or using Novogene’s ultra-low RNA-seq service. Library preparation and sequencing for the initial LCM-seq was performed by the Mellowes Center for Genomic Sciences and Precision Medicine at the Medical College of Wisconsin at 150 bp paired end reads with > 25 million reads per sample. One of the control uninjured samples did not yield enough cDNA library for sequencing and was excluded. Therefore *n* = 2 for uninjured control and *n* = 3 for 3 dpi samples, all males. The raw data is available at the NCBI Sequence Read Archive (SRA) BioProject number PRJNA1139127. Library preparation and sequencing for the morpholino gene knockdown and transgenic overexpression experiments was performed by Novogene Co. using their ultra-low RNA-seq service. Samples were sequenced at > 50 million reads per sample with 150 bp paired end reads. Six experimental groups were included in this study: (1) Wild type, uninjured, untreated; (2) Wild type, 3 dpi (crush); (3) Wild type, control morpholino treated, 3 dpi (crush/cut); (4) Wild type, *Srebf2*-MO2 treated, 3 dpi (crush/cut); (5) *Tg(hsp70l: nSrebf2-2AmCherry*,* cmlc2:EGFP)* + C-MO treated, 3 dpi (crush/cut) + Heat shock; and (6) *Tg(hsp70l: nSrebf2-2AmCherry*,* cmlc2:EGFP)* + *srebf2*-MO2 treated, 3dpi (crush/cut) + heat shock. Each group included *n* = 4 (2 male and 2 female). Raw data is deposited in the NCBI Sequence Read Archive (SRA) BioProject number PRJNA1145997. Gene-specific analysis was performed using Partek™ Flow™ software, v12.3.1 Reads were mapped to zebrafish genome assembly GRCz11 and the ensemble, GRCz11.112.gtf, or Lawson Lab transcriptome annotation files, V4.3.2.gtf, were used [26]. Pre-alignment QA/QC was performed on all samples with the average read quality score > 34.8 for each. Reads were mapped using STAR – 2.7.8a with the default settings on Partek™ Flow™. Post-alignment QA/QC was performed, and alignment rates varied from a low of 54% to a high of 91% suggesting some sample variation was likely created in the library amplification step or due to the limited quantity of starting RNA sample. However, no sample was excluded from further analysis. All QA/QC results are presented in Additional File 2 Table S1. Reads were quantified using the Partek E/M Quantify to Annotation Model. Counts were normalized for each sample using the Median Ratio method for DEseq2. DEseq2 was used to compare differentially expressed genes between treatment groups using the default settings. Gene sets enrichment analysis (GSEA) was performed using WEB-based GEne SeT AnaLysis Toolkit (https://www.webgestalt.org/) [27]. For GSEA parameters, Wikipathway database was used as the functional database; gene sets with less than 5 genes and more than 2000 genes were excluded from the analysis; phenotype permutation of 1000 is used for the GSEA method; gene sets were shown based on normalized enrichment score; and top 10 or 20 up- & down-regulated gene sets were shown as needed. A stringent FDR threshold of < 0.05 was used for all analyses except for morpholino treatment and transgenic line LCM-seq groups where < 0.25 was used. <0.25 is still within the recommended range guidelines for pathway detection using GSEA and was necessitated by nuanced responses in some of our experimental manipulations.

### RNAscope in situ hybridization

Eyes were collected from wild-type zebrafish at three days post crush. The tissue was then fully submerged in 4% paraformaldehyde (PFA) overnight. Post-fixation, the tissues were incubated in 30% sucrose PBS until the tissues sank to the bottom of a 1.5 mL Eppendorf tube, then frozen in Tissue-Tek O.C.T. compound (Sakura Fintech, USA). 14 μm tissue sections were collected using a Microm HM550 cryostat (Thermo Scientific, USA). Every fifth section from the central area of the eye was collected and kept frozen at -80 ℃ before staining. RNAscope Multiplex Fluorescent v2 Kit (Advanced Cell Diagnostics, USA) was used. Probes for *slc17a6b* (Dr-slc17a6b, Cat No. 549001), *srebf2* (Cat No. 1117361-C2), *hmgcra* (Cat No. 1245331-C2), *hmgcrb* (Cat No. 1245341-C3), *sqlea* (Cat No. 1245301-C3), *ldlrb* (Cat No. 1245311-C1), and *npc1* (Cat No. 1245351-C3,) were designed and purchased from Advanced Cell Diagnostics, USA. Images were acquired with Zeiss LSM 980 confocal microscope. Images were analyzed in ImageJ software to count puncta per cell in the retina GCL [28].

### RGC retrograde labeling

To assay the efficiency of MO delivery into RGCs, *Tg(-17.6isl2b: GFP)*^*zc7*^ [29] transgenic zebrafish, which has GFP expressed in the RGC, was used as a reporter line. 3’-Lissamine conjugated standard control-MO (Gene Tools, USA) was delivered using the same MO treatment procedure mentioned above. Then, the injured retina was harvested at 3 h post-injury. After fixing in 4% PFA at 4 ℃ overnight, the retina was flat mounted using VECTASHIELD anti-fade mounting media (H-1000-10, Vector Laboratories, USA) within a 0.12 mm spacer (S24735, Life Technologies). GFP and Lissamine were imaged using Oxford Instruments Andor BC43. 4–5 images near the optic nerve head at 40x, 0.5 mm z-step were taken for analysis. For total RGC quantification in the GCL, retrograde labeling from the transected ON was performed as above and 3000 MW rhodamine dextran (D3308, ThermoFisher) was applied at the time of transection.

### RNA isolation and quantitative real-time PCR

For quantitative real-time PCR, retina from injured and control eyes were dissected. Total RNA was isolated using TRIzol Reagent (15596026, Invitrogen, USA) according to the manufacturer’s instructions. Total RNA (200 ng) was reverse transcribed using the SuperScript III reverse transcriptase kit (Invitrogen, USA). *srebf2*, *hmgcrb*, *pggt1b*, *fntb*, *sqlea*, and *ldlrb* were amplified using SYBR Green PCR Master Mix (Applied Biosystems, USA). Primers *gapdh*-for (5′-ATGACCCCTCCAGCATGA-3′) and *gapdh*-rev (5′-GGCGGTGTAGGCATGAAC-3′), *srebf2*-for (5′- GATTCTGGAGACACAGGAAAC-3′), *srebf2*-rev (5′- CTCTGGATAACACTGACAGACAC-3′), *hmgcrb*-for (5′- CCTGTTAGCCGTCAGTGGA-3′), *hmgcrb*-rev (5′- TCTTTGACCACTCGTGCCG − 3′), *ldlrb*-for (5′- TTCGTCTGGCCAATCACACA-3′), *ldlrb*-rev (5′- GGTCATGTGATCCAGCTCGT-3′), *sqlea*-for (5′- GCGGAAATCCTCTCACTCGT-3′), *sqlea*-rev (5′- GCTCGTGGTATTGTGAGCCA-3′), *fntb*-for (5′- CGGAGGAGGCCTACAATGTC-3′), *fntb*-rev (5′- GTCCCATCAAACAGGGTGGG-3′), and *pggt1b*-for (5′- TCTGCTTCATGCTGGACGAC-3′), *pggt1b*-rev (5′- GTAGCAGGTGTCCACAGGTT-3′) were used. The Bio-Rad CFX96 Touch Real-Time PCR System was used. Cq values were detected using 35 amplification cycles (30s of activation & denaturation at 90 ℃, 35 cycles of 5s 90 ℃, 10s 52 ℃, and 15s 60 ℃, end with 10s 90 ℃ for final extension). A melting curve was used to determine PCR efficiency and specificity. mRNA expression levels were quantified using the delta-delta Ct method [30] with *gapdh* as the reference gene. All reactions were performed in triplicate, and at least three independent biological samples were analyzed per experimental group.

### Quantification of optic nerve and optic tectum reinnervation

Regenerating axons from the retina toward the optic nerve and optic tectum were visualized by using *Tg(3.6fgap43:GFP)*^*SA1*^ zebrafish [31]. To visualize optic nerve regeneration, fish were euthanized by overdose of tricaineat 3 days post optic nerve crush. After 4% PFA fixation overnight, the optic nerves from the optic nerve head to the rostral optic nerve tracts including the chiasm was dissected out in a single piece and mounted using VECTASHIELD anti-fade mounting media (H-1000-10, Vector Laboratories, USA) within a 0.12 mm spacer (S24735, Life Technologies) to prevent over-compression. Then, optic nerve images were taken at x 4 objective lens using a Nikon Eclipse 80i microscope. Quantification was done by acquiring the mean GFP intensity in 50 μm steps from the injury site to the optic chiasm. Regeneration index calculation: $\:(F-{F}_{0})/{(F}_{pre}-{F}_{0})$, where *F* is the mean GFP intensity at each 50 μm range, *F*_*pre*_ is the mean GFP intensity of the injured optic nerve prior to the injured site close to the optic nerve head, and *F*_*0*_ is the mean GFP intensity of the whole uninjured optic nerve.

For optic tectum regeneration measurement, at seven days post nerve crush, fish were euthanized, and brains were dissected and fixed overnight in 4% PFA. After rinsing in PBS and embedding in 4% agarose (in PBS), 50-μm thick coronal vibratome (Leica VT1000S, Germany) sections were made. Brain sections throughout the optic tectum (17–24 sections per fish) were collected and mounted with VECTASHIELD anti-fade mounting media (Vector Laboratories, USA). Optic tectum images were acquired with a Nikon Eclipse 80i microscope with pco.panda 4.2 camera at × 4 objective lens. Tectal reinnervation was quantified by calculating the change in F/F ratio of fluorescent density from the stratum fibrosum et griseum superficiale (SFGS) and stratum opticum (SO) of the optic tectum from the crushed side over the uninjured side (Figure S4). For better spatial resolution, the whole optic tectum was separated into 3 radial regions (dorsal, medial, and ventral). Per fish, tectum reinnervation was analyzed in sequential order from rostral to caudal and normalized to 100% of tectal length to adjust for variability in brain sizes from fish to fish. In all experiments, 5–7 fish were included in each group.

### Dorsal light response (DLR) assay

The DLR assay was performed as described in Diekmann et al. [32] with slight modification. After ON injury, fish swim at a tilted angle(∼ 8–12°) with the blind eye up and the sighted eye down [32, 33]. This is an attempt to equilibrate the light entering each eye which helps define the vertical position in this free swimming species. As vision recovers in the blind eye the degree of tilt decreases proportionally. At various times after optic nerve injury, fish were placed into a 5.5 × 26.5 × 15 cm container with 400 ml water. After 5 min adaptation, they were recorded on video for 2 min, making sure to capture at least five straight swims directly towards the camera. The videos were analyzed frame by frame, and still pictures were taken if the whole body of the fish was positioned straight toward the camera. The angle between the fish body position and the horizon was then determined using ImageJ. At least five different pictures were analyzed per fish and time point to calculate the mean tilting angle. For each group, at least 5 fish were used.

### GCL cell count

Retina from the injured eyes was collected after the last DLR recording (21 dpi). After fixing in 4% PFA at 4 ℃ overnight, DAPI (62248, Thermo-Fisher) nucleus staining was performed. The retina was flat mounted using VECTASHIELD anti-fade mounting media (H-1000-10, Vector Laboratories, USA) within a 0.12 mm spacer. Then, 4–5 images from the central (near the optic nerve head) and peripheral (near the edge of the retina) were taken using LSM 980 (Zeiss, Germany) under 40x, 1 mm z-step for the nucleus count. The cell density in the GCL of fish changes during the lifespan due to their indeterminant growth and retinal stretching as the eye grows in size [34, 35]. We were careful to use age and size matched fish for each experiment, usually siblings raised in adjacent tanks. We do observe slight differences in GCL cell densities between experiments likely due to different aged adult fish and stocking densities affecting overall adult fish and therefore eye size.

### Statistical analysis

All data are represented as mean ± SEM, and the value of n represents the number of animals used per condition. All statistical tests were performed using Graphpad Prism 10. In all cases, raw data were tested for normal distribution using the Kolmogorov-Smirnov normality test. The variance between groups was checked via Brown-Forsythe’s test for equality of variances. To evaluate a difference in puncta per cell in the GCL, a two-tailed Student’s t-test was performed. To compare optic tectum reinnervation between control and treated groups, a two-way ANOVA was performed if the data showed a normal distribution and variances between groups were homogeneous. A Fisher’s LSD *post-hoc* test was performed, and only the *p* values indicating a significant difference between two values/conditions are shown. *p* < 0.05 was considered statistically significant. For DLR, the significance of intergroup differences was evaluated using Repeat-Measurements Two-Way ANOVA with Bonferroni *post hoc* tests.

## Results

### The cholesterol synthesis pathway is upregulated in the GCL during ON regeneration

**Fig. 1:**
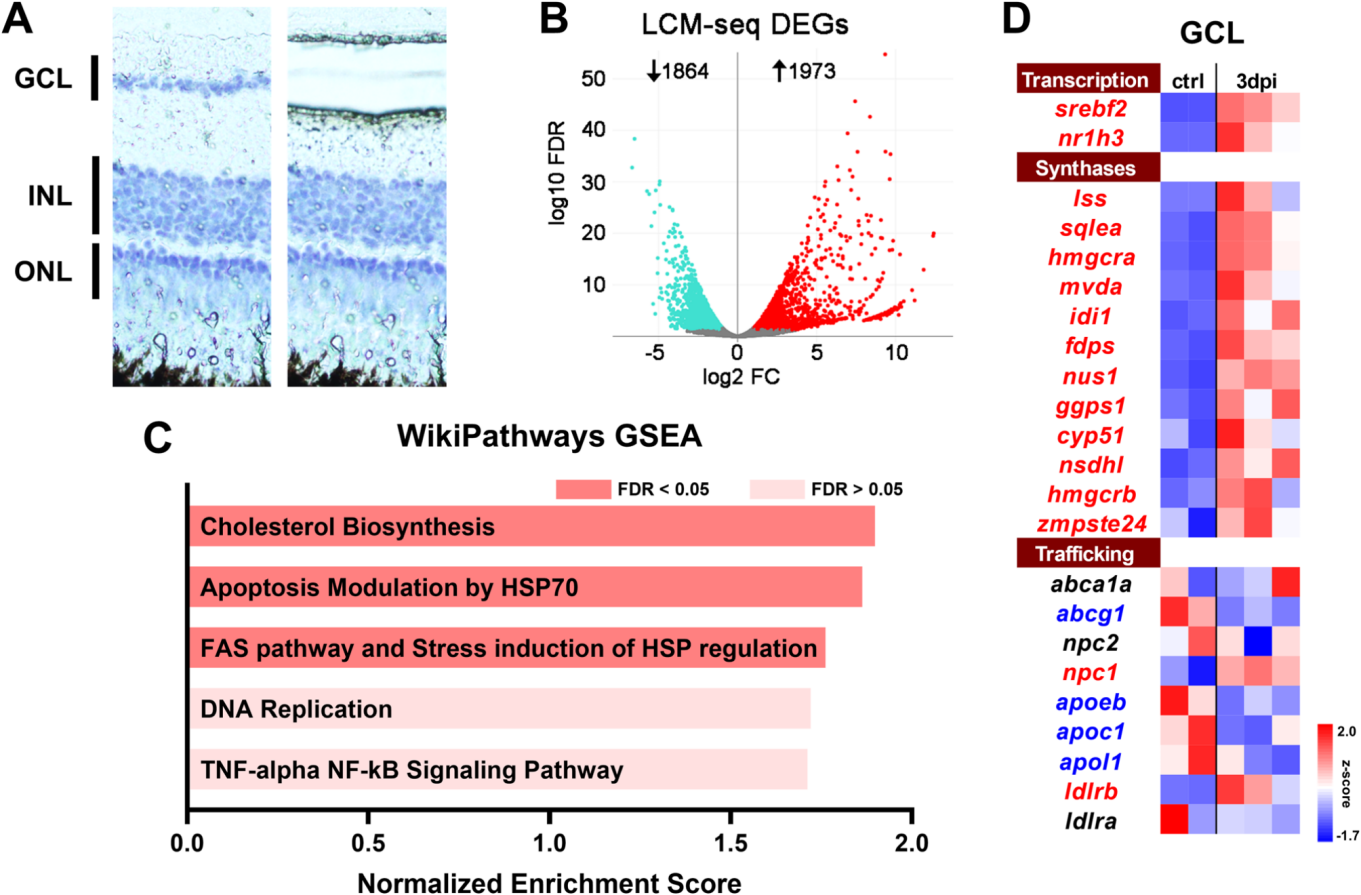
LCM-seq analysis of 3 dpi retinal ganglion cell layer (GCL) identifies upregulation of the cholesterol synthesis pathway. (**A**) Representative image from pre- and post-GCL LCM retina section. (**B**) Volcano plot (|FoldChange| > 1, FDR < 0.05) of differentially expressed gene from 3 dpi (*n* = 3) vs. uninjured GCL (*n* = 2). (**C**) GSEA of DEGs genes based on zebrafish WikiPathways. (**D**) Heatmap of cholesterol-related transcriptional regulators, cholesterol synthases, and cholesterol trafficking genes of 3 dpi vs. uninjured from GCL. Gene names in red or blue indicates *p* < 0.05 by DEseq2 in 3 dpi group versus control where red represents upregulated genes and blue represents downregulated genes

**Fig. 2:**
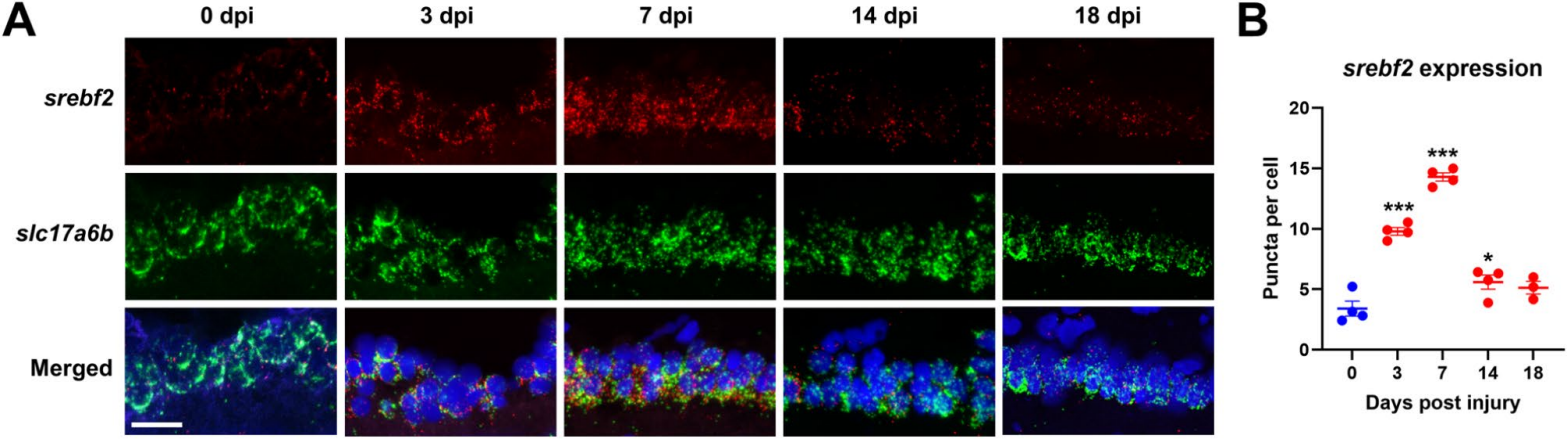
*srebf2* is upregulated in RGCs during axon regeneration. (**A**) Representative images of GCL *srebf2* expression at various time points post nerve injury with *slc17a6b* as RGC marker. (**B**) Quantification of GCL *srebf2* expression. * *p* < 0.05, and *** *p* < 0.001 compared with 0 dpi (uninjured). Scale bar = 20 μm. One-way ANOVA with Bonferroni post hoc was used as statistical analysis, *n* = 4 for each group

To understand the general transcriptomic changes during ON regeneration from an enriched population of RGCs, we performed LCM of the GCL. The GCL of teleost fish, including zebrafish, is reported to have many fewer displaced amacrine cells than mammals [36, 37]. To confirm this we used retrograde tracing and the RGC specific *isl2b: GFP* transgenic line [29] to estimate that ∼ 90% of the cells in the GCL are RGCs (Figure S1). Total mRNA was collected from the GCL from control (*n* = 2) and 3 days (*n* = 3) post optic nerve crush injury retinas for RNA-seq (Fig. 1A). This time point corresponds to robust axon regrowth throughout the ON and approaching the optic chiasm. Hierarchical clustering of these samples by treatment and based upon normalized gene expression clearly segregated the control uninjured and 3 dpi groups (Figure S2). We found that there are 1973 genes upregulated and 1864 genes downregulated (FDR < 0.05,|log2 Fold Change| > 1) (Fig. 1B, Table S2). Then we performed gene set enrichment analysis (GSEA) [38] using the WikiPathways database [39], we found that the chol biosynthesis pathway is the top-upregulated pathway (Fig. 1C, Figure S3). We next curated a gene list, heatmap in Fig. 1D, comprising genes involved in chol biosynthesis in the WikiPathways database, which we then supplemented with chol-related transcription factors (TFs), *srebf2* and *nr1h3*, and chol trafficking genes identified in the literature [22, 40]. In the GCL, we observed upregulation of both chol biosynthesis genes and chol-related TFs (Fig. 1D). Upregulated genes included those in the mevalonate synthesis pathway which transforms Acetyl-CoA into squalene and the post squalene pathway that completes chol synthesis (Figure S4). To understand the role of the entire cholesterol biosynthesis pathway in successful axon regeneration, we initially decided to focus on the master cholesterol-related TF of this pathway, *srebf2*. Srebf2 activity is activated when chol is low in the endoplasmic reticulum. This basic helix-loop-helix (bHLH) transcription factor (TF) activates genes, including nearly all in the chol synthesis pathway, through specific DNA binding at the sterol regulatory element (SRE) [41, 42]. To examine changes in *srebf2* expression in RGCs over the course of ON regeneration, we performed in situ hybridization of retinal sections at different time points post ON injury. *slc17a6b* probe (previously known as *vglut2a*) was used to identify RGCs in the GCL, and *srebf2* probe was used to detect *srebf2* expression (Fig. 2A). *slc17a6b* is not among the differentially expressed genes at 3 dpi. Quantification of mRNA puncta demonstrated that *srebf2* is upregulated at 3 dpi until 14 dpi compared with uninjured (0 dpi) expression (Fig. 2B). We did not observe changes in *srebf2* expression in the other retinal layers at the timepoints examined.

These data indicate the cholesterol biosynthesis pathway is transcriptionally upregulated following optic nerve injury, and its master regulator TF – *srebf2* is upregulated throughout the axon regeneration process.

### srebf2 loss-of-function inhibits axon regeneration in the optic nerve and optic tectum

To perform loss-of-function experiments, we tested the *srebf2* inhibitor—fatostatin [43] and two independent *srebf2* morpholino antisense (MO) to inhibit translation (*srebf2*-MO1) or cause mis-splicing (*srebf2*-MO2) to knockdown *srebf2* in the RGCs. The efficiency of MO delivery to RGCs was determined by adding 3’ lissamine-tagged control (Ctrl) MO to the severed nerve and counting the lissamine-positive RGCs (Figure S5). The result showed that around 90% *Tg(-17.6isl2b: GFP)*^*zc7*^ positive RGCs were also labeled with lissamine MO, showing around 90% MO delivery efficiency into RGCs, similar to a previous report [4]. To assess the binding specificity and duration of *srebf2*-MO efficacy, we delivered *srebf2*-MO2 into RGCs and collected whole retina samples to detect exon mis-splicing by RT-PCR. Mis-splicing was only detectable in *srebf2*-MO2 treated groups and lasted at least 14 days (Figure S6).

**Fig. 3:**
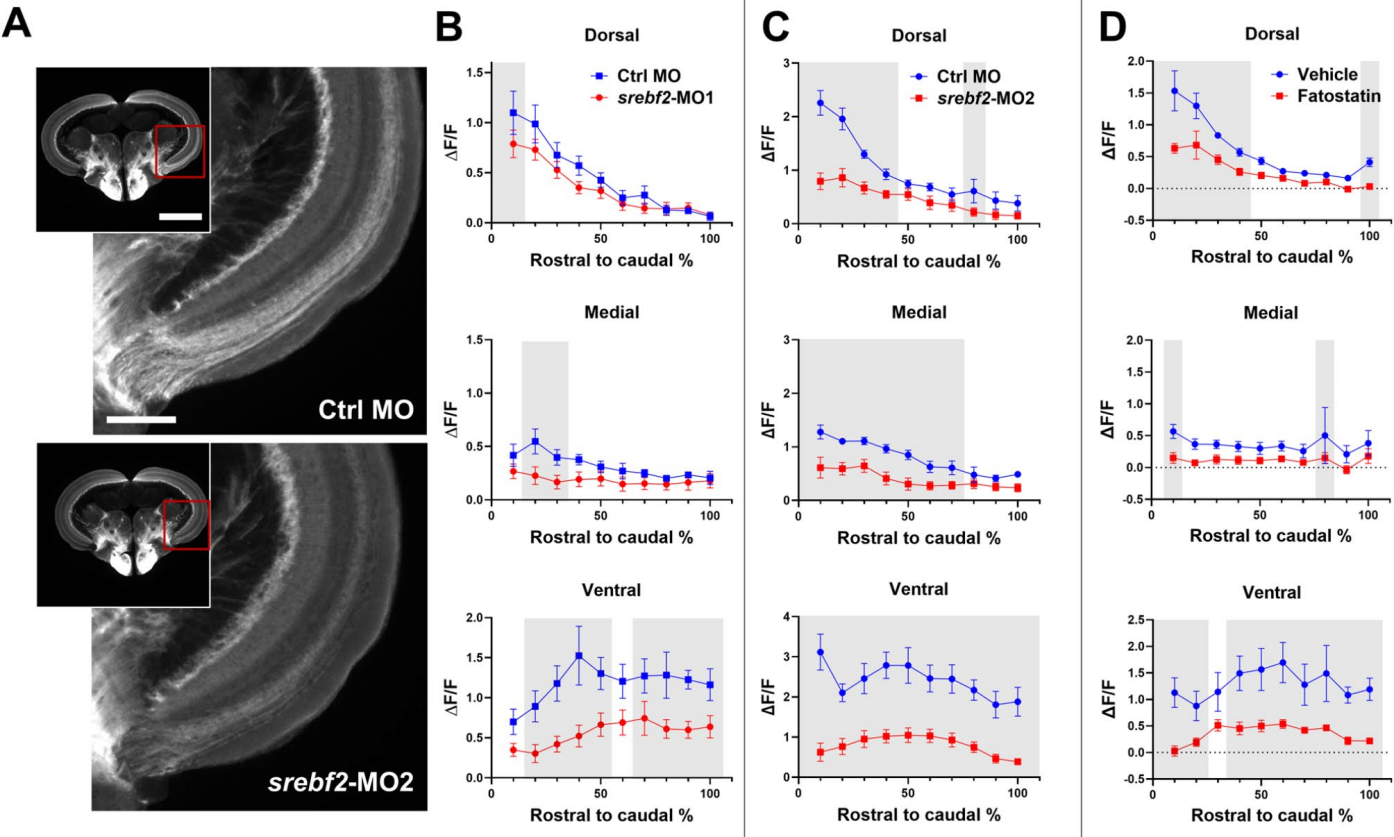
Srebf2 loss-of-function inhibits axon regeneration in the optic tectum at 7 dpi. (**A**) Representative images of tectum and higher magnification of medial-ventral tectum of vehicle or fatostatin treated zebrafish. (**B**) Quantification of tectum regeneration at dorsal, medial, and ventral region after *srebf2*-MO1 treatment. (**C**) Quantification of tectum regeneration at dorsal, medial, and ventral region after *srebf2*-MO2 treatment. (**D**) Quantification of tectum regeneration at dorsal, medial, and ventral region post fatostatin treatment. Grey boxes indicate *p* < 0.05 compared with Ctrl MO or Vehicle at the corresponding rostral to caudal distance by two-way ANOVA with Fisher’s LSD post hoc test. Scale bar = 500 and 200 μm, *n* = 5–6 for each group

To determine whether *srebf2* loss-of-function inhibits axon regeneration after optic nerve injury, optic tectum regeneration assay at 7 dpi was performed. 7 dpi represents the maximum axon regenerative response as measured in the optic tectum [44, 45] and allows for detection of delayed or accelerated regeneration. The *Tg(3.6fgap43:GFP)*^*SA1*^ transgenic zebrafish were used to detect regenerating axons by GFP intensity. This assay measured the ΔF/F ratio of the SO/SFGS layer on serial sections of the optic tectum (Fig. 3A, Figure S7). By segmenting the measurements into dorsal, medial, and ventral regions of the optic tectum, we have a sensitive measure of how the axon regeneration pattern in the tectum is influenced by *srebf2* loss-of-function. We find that either fatostatin treatment or each *srebf2*-MOs (Fig. 3B-D) significantly reduce axon regeneration into the optic tectum at 7 dpi. The most robust effect is seen in the ventral optic tectum across the full distance of the tissue in each treatment group. This is likely due to the perpendicular plane of the section through the ventral optic tract while the dorsal tract is sectioned at an angle and the medial tectum is the last region to be reinnervated during ON regeneration.

We also assayed the optic nerve regeneration in the ON by measuring *Tg(3.6fgap43:GFP)*^*SA1*^ intensity in the ON at 3 dpi. Like what we observed in the optic tectum, both *srebf2*-MOs and fatostatin treatment decreased regeneration in the ON compared to the control-MO or vehicle-treated group (Figure S8). Together, these data show that *srebf2* loss-of-function reduces axon regeneration in both the optic nerve and optic tectum.

### Srebf2 loss-of-function delays functional vision recovery without causing loss of RGCs

**Fig. 4:**
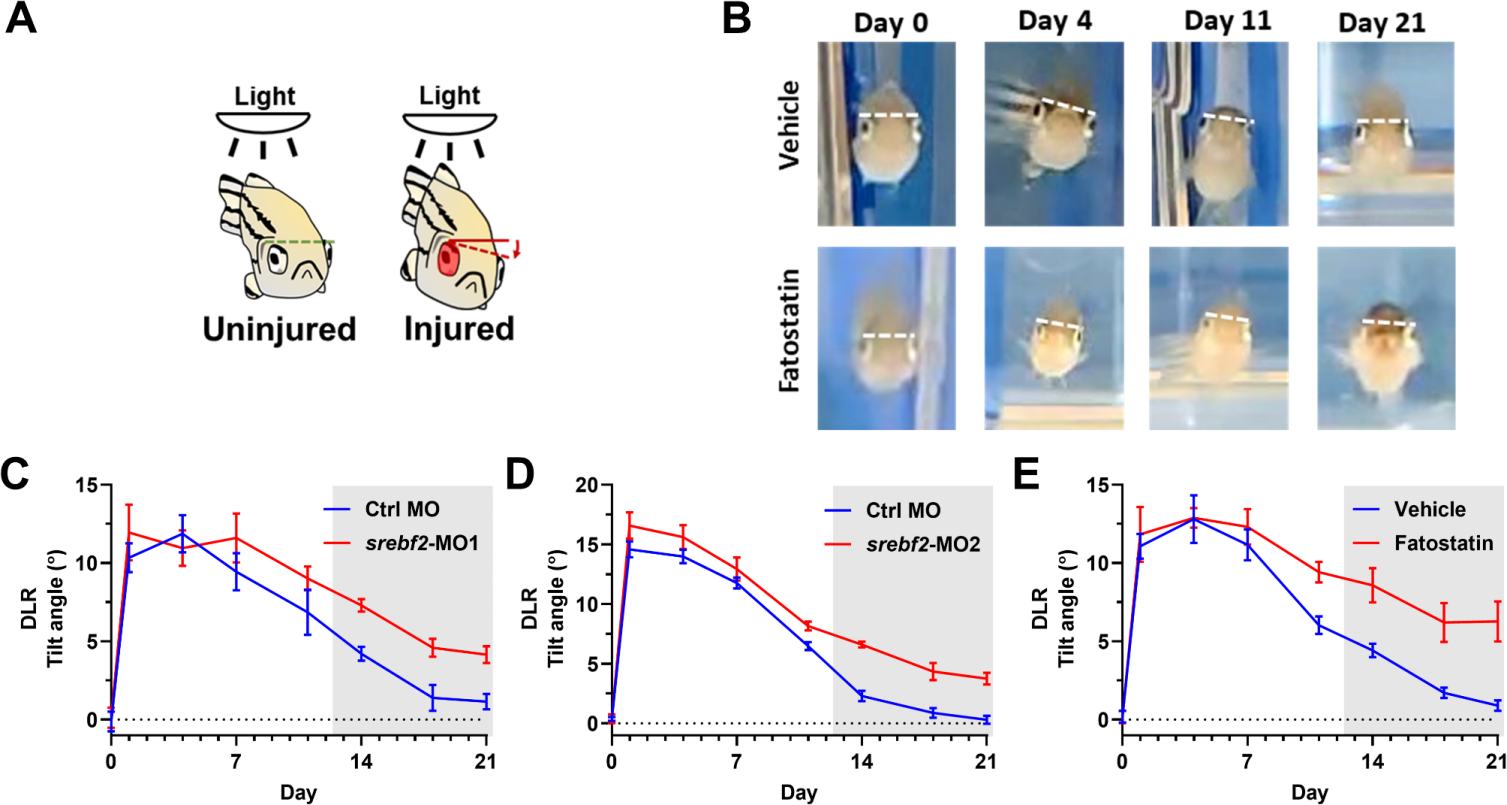
Srebf2 loss-of-function delays dorsal light response (DLR) recovery. (**A**) Schematic of the DLR. (**B**) Representative images of the DLR after nerve injury with vehicle or fatostatin treatment over time. (**C**) Quantification of DLR recovery after control MO (Ctrl MO) or *srebf2*-MO1 treatment. (**D**) Quantification of DLR recovery after control MO (Ctrl MO) or *srebf2*-MO2 treatment. (**E**) Quantification of DLR recovery after Vehicle or fatostatin treatment. Grey boxes indicate *p* < 0.05 compared with Vehicle or Ctrl MO at corresponding days post-injury by two-way ANOVA with Bonferroni post hoc test. The dotted line is a 0-degree tilt angle observed at day 0, *n* = 5–6 for each group

To measure vision loss and recovery, we used the dorsal light response (DLR). Fish tend to keep their back oriented toward a source of light to maintain a vertical position in the water. In part, the fish accomplishes this by equilibrating the amount of light exposure to each eye. This behavior persists even after monocular optic nerve injury, resulting in a tilted angle of swimming where the uninjured eye is tilted away from the light source to balance the light intensity perceived through each eye (Fig. 4A). As vision recovers the fish’s posture returns to vertical. Both fatostatin and *srebf2*-MOs treatments resulted in delayed tilting angle recovery, starting from 14 dpi and lasting until 21 dpi (Fig. 4B). In contrast, the vehicle or control-MO treated group achieved full recovery within this timeframe (Fig. 4C-E). After the final DLR test at 21 dpi, we harvested the retinas for flat-mount GCL cell counts. The result showed that neither fatostatin nor *srebf2*-MOs treatment led to significant cell number change, which indicates that the delayed DLR recovery is not caused by RGC cell loss (Figure S9).

### Constitutively active *srebf2* overexpression rescues *srebf2* loss-of-function

**Fig. 5:**
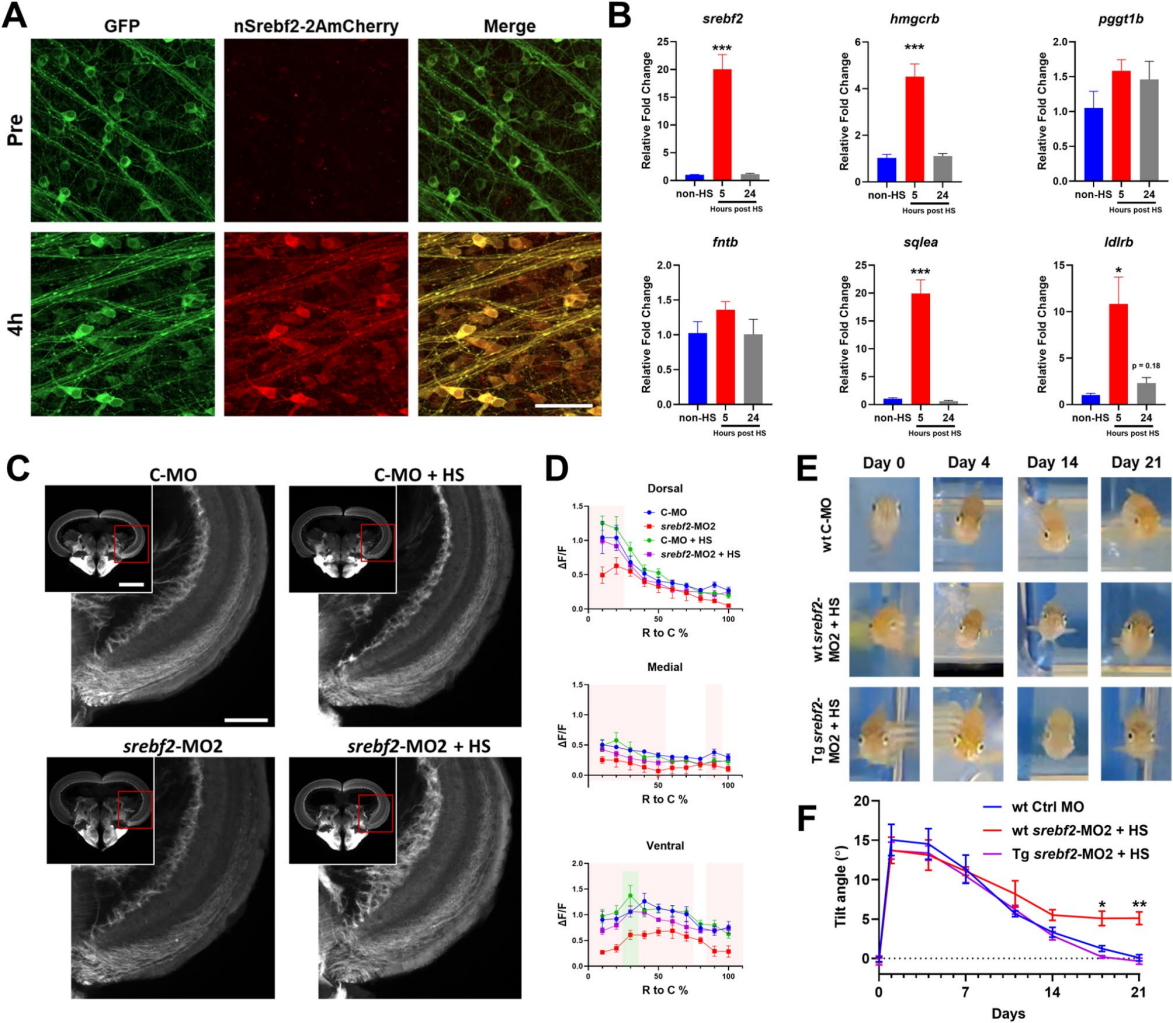
Conditional expression of constitutively active *srebf2* rescues loss-of-function but does not accelerate regeneration. (**A**) mCherry expression in whole mount *Tg(hsp70l: nSrebf2-2AmCherry*,* cmlc: GFP)* zebrafish retina GCL before heat shock (Pre) and 4-hours post heat shock (4 h). (**B**) Expression of selected mev/chol synthesis pathway genes in post heat shock retina measured by qRT-PCR by one-way ANOVA with Bonferroni post hoc test, *n* = 4 for each group. (**C**) Representative images of axon regeneration into the optic tectum after treatment with control MO (C-MO) or *srebf2*-MO2 with and without heat shock rescue (+ HS) by *Tg(hsp70l: nSrebf2-2AmCherry*,* cmlc: GFP)*. (**D**) Quantification of regeneration into the optic tectum. Red boxes represent *p* < 0.05 in C-MO vs. srebf2-MO2 group at the corresponding rostral to the caudal region; green boxes represent *p* < 0.05 in C-MO vs. C-MO + HS by two-way ANOVA with Fisher’s LSD post hoc test, no other comparisons were statistically significant, *n* = 5–6 for each group. (**E**) Representative images of the DLR on various days post nerve injury with C-MO, *srebf2*-MO2, and *srebf2*-MO2 plus transgenic heat shock rescue. (**F**) Quantification of the DLR in each treatment group over time by two-way ANOVA with Bonferroni post hoc test, *n* = 5–6 for each group. + HS indicates daily heat shock treatment starting at day 0. Scale bar in A = 50 μm. Scale bar in C = 500 μm and 200 μm. * *p* < 0.05, ** *p* < 0.01, *** *p* < 0.001

To determine if *srebf2* is sufficient to accelerate axon regeneration, we generated a *Tg(hsp70l: nSrebf2-2AmCherry; cmlc: GFP)* line to overexpress constitutively active nuclear *srebf2* after heat shock treatment (Figure S10). The nuclear *srebf2* consists of the bHLH domain of *srebf2*, which directly binds to the SRE and activates transcription without being affected by fatostatin or *srebf2*-MOs [43, 46]. To validate the function of the transgenic line we heat shocked embryos (Figure S10) and adults (Fig. 5A, Figure S10). We observed increased mCherry expression through the lens and flat mount retina GCL at 4 h post heat shock (Fig. 5A, Figure S10) as well as whole embryos (Figure S8). To confirm constitutively active *srebf2* activity, we performed whole retina qRT-PCR and tested Srebf2-regulated genes, endogenous *srebf2* itself, *hmgcrb*, *sqlea*, and *ldlrb* are upregulated at 4 h but not 24 h post heat shock (Fig. 5B). These data demonstrate that *Tg(hsp70l: nSrebf2-2AmCherry; cmlc: GFP)* is functioning as designed in the retina to upregulate Srebf2 target genes.

We first tested if constitutively active *srebf2* expression itself could accelerate axon regeneration at 7 dpi. To control for the heat shock treatments, we tested if *srebf2*-related gene expression was affected by heat shock alone. We found that *fntb* and *sqlea* were slightly downregulated after heat shock alone (Figure S11), but the other genes tested (*srebf2*, *hmgcrb*, *pggt1b*, and *ldlrb*) were unaffected and *Tg(hsp70l: nSrebf2-2AmCherry; cmlc: GFP)* heat shock induced strong upregulation of these genes. Showing that small effects caused by heat shock alone are overcome by the robust effects of the transgene. Then, we did an ON regeneration assay in the *Tg(3.6fgap43:GFP)*^*SA1*^ line with daily heat shock and confirmed that axon regeneration was no different than non-heat shock control (Figure S11). Similarly, daily heat shock of *Tg(hsp70l: nSrebf2-2AmCherry; cmlc: GFP)* treated with control MO (Fig. 5C, D) versus control MO only (Fig. 5C, D) did not largely affect the axon regeneration with only a slight increase at a single location in the ventral optic tectum. This suggests that *srebf2* activation alone is insufficient to accelerate ON regeneration in zebrafish.

To confirm the specificity of the Srebf2 loss-of-function effect on ON regeneration, we determined if daily heat shock induced *srebf2* overexpression could rescue the inhibitory effect of *srebf2*-MO2 treatment. Using *Tg(hsp70l: nSrebf2-2AmCherry; cmlc: GFP)* line with *Tg(3.6fgap43:GFP)*^*SA1*^ background we found that, consistent with the previous result, *srebf2*-MO2 inhibited axon regeneration and that heat shock induced nuclear *srebf2* overexpression was sufficient to rescue the *srebf2*-MO2 effect (Fig. 5C, D). We then evaluated if nuclear *srebf2* overexpression can rescue *srebf2*-MO2 delayed vision recovery by the DLR test. *Tg(hsp70l: nSrebf2-2AmCherry; cmlc: GFP)* zebrafish were treated with *srebf2*-MO2 and given daily heat shocks (Fig. 5E, F). We found that nuclear *srebf2* overexpression was sufficient to recover the DLR deficit created by srebf2 gene knockdown (Fig. 5E, F, and S10). We also noted that like the result from the ON regeneration assay, nuclear *srebf2* overexpression did not accelerate DLR recovery. After the final DLR test, we harvested the retinas for GCL cell count and found there was no change in RGC abundance (Figure S12). These results suggest that nuclear *srebf2* overexpression is sufficient to reverse the inhibitory effect from *srebf2* loss-of-function but not able to accelerate regeneration.

### *srebf2* regulates axon regeneration through the mevalonate synthesis pathway

**Fig. 6:**
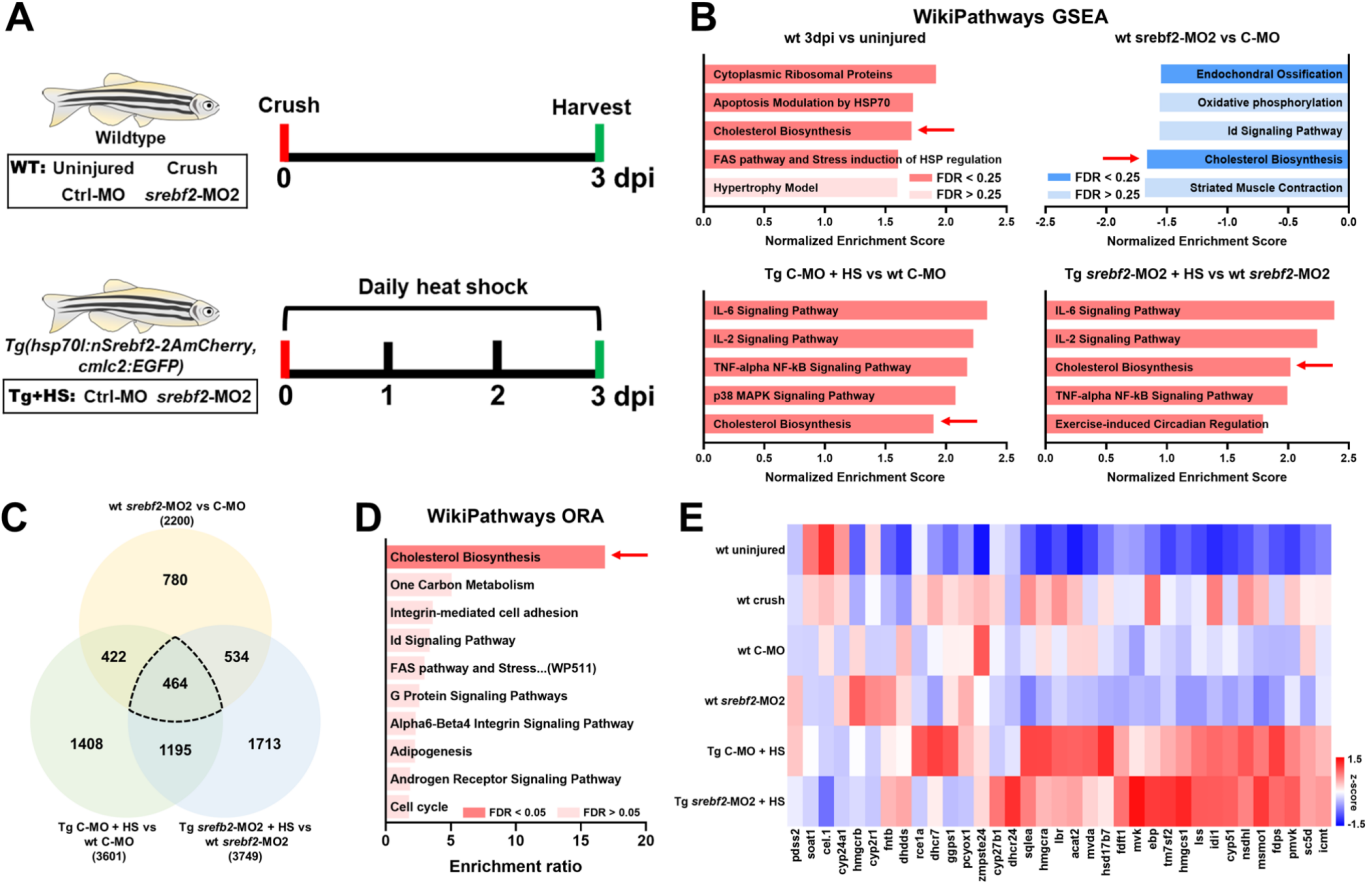
The Cholesterol Biosynthesis pathway is coordinately regulated in the retinal GCL by *srebf2* knockdown or constitutively active overexpression at 3 dpi as measured by LCM-seq. **A**) The six experimental groups being compared by LCM-seq and the heat shock treatment regimen. *N* = 4 fish per treatment group, two males and two females. **B**) The mev/chol pathway is up regulated 3 dpi, down regulated by *srebf2*-MO2 treatment, and up regulated by transgenic (Tg) constitutively active *srebf2* over expression in control MO and *srebf2*-MO2 treated groups (FDR < 0.25). GSEA based on zebrafish Wikipathway. **C**) Venn diagram for differentially expressed genes (|FoldChange| ≥ 1.5) in the different comparison groups identifies 464 common genes. **D**) WikiPathways over-representation analysis of the 464 common genes in C enriches for the mev/chol pathway. **E**) Heatmap of mev/chol synthesis gene expression in each experimental group. 3dpi, uninjured, C-MO, and *srebf2*-MO2 groups used wt zebrafish; Tg C-MO + HS and Tg *srebf2*-MO2 + HS used *Tg(hsp70l: nSrebf2-2AmCherry*,* cmlc: GFP)* zebrafish with daily heat shock (HS)

To determine the downstream transcriptional effects *srebf2* activity during ON regeneration, we performed LCM-seq on the GCL at 3 dpi on uninjured or MO treated control animals, *srebf2* loss-of-function using srebf2-MO2, or *srebf2* overexpression using the *Tg(hsp70l: nSrebf2-2AmCherry; cmlc: GFP)* line (Fig. 6A). Hierarchical clustering of these samples by transcriptome-wide gene expression grouped them broadly by uninjured versus injured/regenerating and secondarily separated the injured/regenerating samples by heat shock versus no heat shock (Figure S13). Srebf2 experimental manipulation by MO did not segregate samples into clusters based upon transcriptome-wide clustering. We separated samples into different comparison groups (wt 3dpi versus wt uninjured, wt C-MO versus wt *srebf2*-MO2, and Tg C-MO + HS versus Tg *srebf2*-MO2 + HS) and performed WikiPathways GSEA (Fig. 6B and S14). Here we adjusted the GSEA FDR significance threshold to < 0.25 versus the more stringent < 0.05 (Fig. 6B) due to the more nuanced response to the MO treatment or nuclear Srebf2. This threshold is acceptable based upon the GSEA user guide (www.gsea-msigdb.org) for general conclusions. Again “Cholesterol Biosynthesis” was among the highly enriched pathways between the wt 3dpi versus uninjured groups supporting our initial observation presented in Fig. 1. We found that the chol biosynthesis pathway is also one of the top 5 regulated pathways among the other comparison groups (Fig. 6B, Figure S14). Chol biosynthesis was enriched in upregulated genes at 3 dpi, after *Tg(hsp70l: nSrebf2-2AmCherry; cmlc: GFP)* heat shock, and after *Tg(hsp70l: nSrebf2-2AmCherry; cmlc: GFP)* heat shock in *srebf2*-MO2 treated fish, while it was downregulated in *srebf2*-MO2 treatment alone. To understand the common pathways that are differentially regulated among all *srebf2* manipulation groups, we filtered for the genes that have greater than 1.5 of absolute fold change value among all three groups (464 genes) and ran over-representation analysis (ORA) using the WikiPathways database. We found that the cholesterol biosynthesis pathway has the highest enrichment ratio (Fig. 6C-D, Table S3). To demonstrate this observation at the individual gene level, we combined gene sets related to cholesterol biosynthesis/metabolite from WikiPathways, the Kyoto Encyclopedia of Genes and Genomes (KEGG) pathway, and the Reactome pathway databases. Subsequently, we generated a heatmap of chol biosynthesis pathway genes (Fig. 6E). We found that the wt control-MO treated GCL had increased cholesterol synthesis genes similar to wt 3 dpi levels when comparing to the wt uninjured group, and wt *srebf2*-MO2 treatment decreased this trend, heat shock inducible nuclear *srebf2* overexpression dramatically increased most chol synthesis genes with both control-MO treatment and *srebf2*-MO2 treatment. These findings suggested that the chol biosynthesis pathway is downstream of *srebf2* in RGCs and likely to be mediating the observed effects on ON regeneration.

**Fig. 7:**
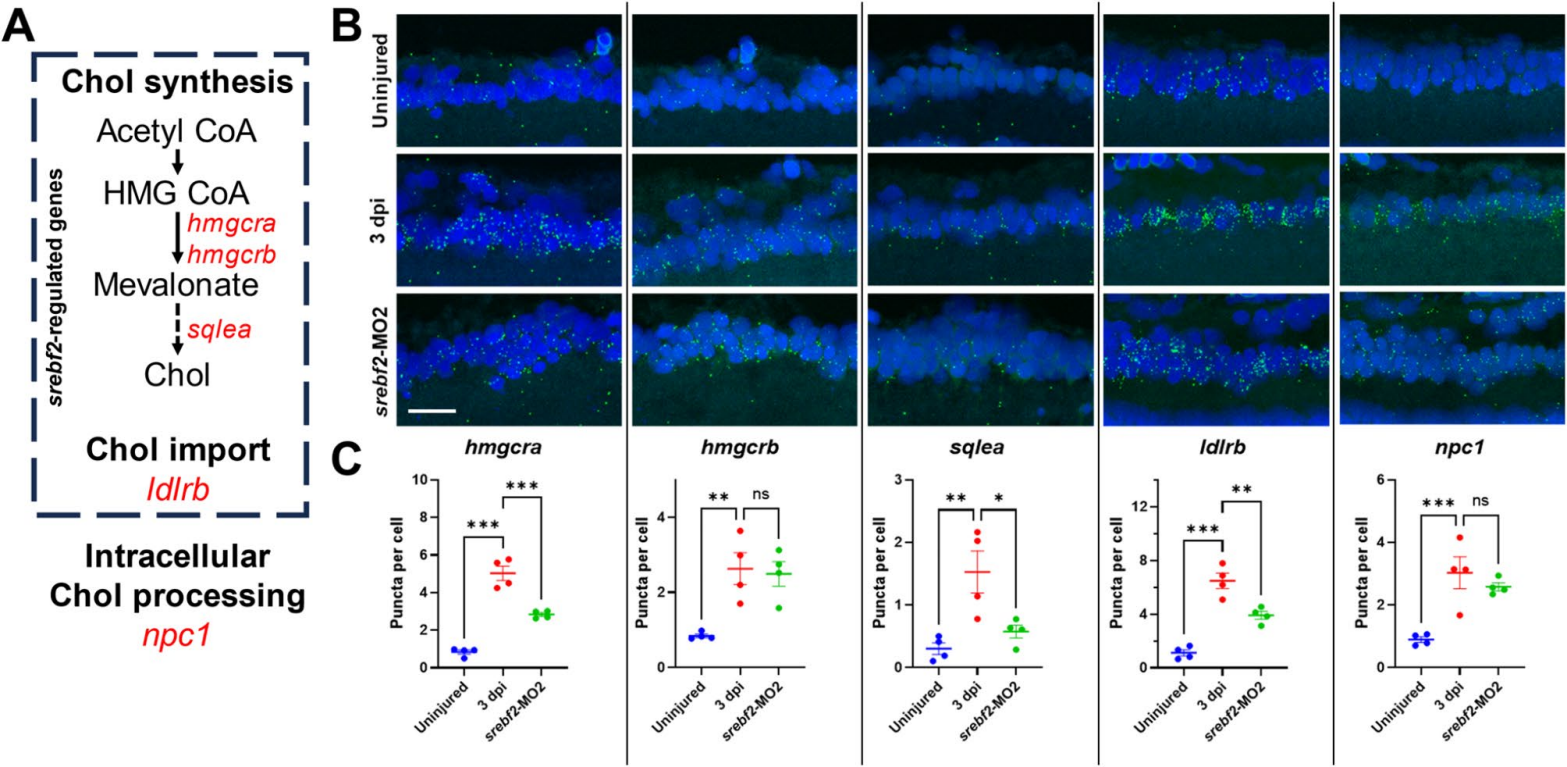
GCL mev/chol synthesis genes are dependent on *srebf2* activity for expression at 3 dpi. (**A**) Location of the tested genes in the mev/chol pathway. (**B**) In situ hybridization detection of each gene in uninjured, optic nerve injury, and srebf2-MO2 treated 3 dpi retinal GCL. (**C**) Quantification of GCL mRNA transcript abundance by one-way ANOVA with Bonferroni post hoc test, *n* = 4 for each group. Scale bar = 20 μm. * *p* < 0.05, ** *p* < 0.01, and *** *p* < 0.001

To confirm our LCM-seq measured effects of ON injury and *srebf2* loss-of-function, we performed in situ hybridization for *hmgcra*, *hmgcrb*, and *sqlea* (Fig. 7A). The *hmgcra* and *hmgcrb* genes encode the rate-limiting enzymes for the mevalonate synthesis pathway, which is upstream of chol synthesis and protein prenylation (Fig. 7A). The *sqlea* gene encodes the key enzyme of chol synthesis downstream of the mevalonate pathway. We used *ldlrb* as a positive control and *npc1* as a negative control for *srebf2* regulation [47, 48]. Quantification of mRNA puncta showed that all five genes are upregulated in the GCL after optic nerve injury (Fig. 7B, C). No change in other retinal layers were noted. *srebf2*-MO2 treatment significantly attenuated this effect on *hmgcra*, *sqlea*, and *ldlrb* expression while *hmgcrb* and *npc1* did not change (Fig. 7B, C). This confirms the LCM-seq data that key genes in the mev/chol synthesis pathway are transcriptionally downstream of *srebf2* during ON regeneration.

**Fig. 8:**
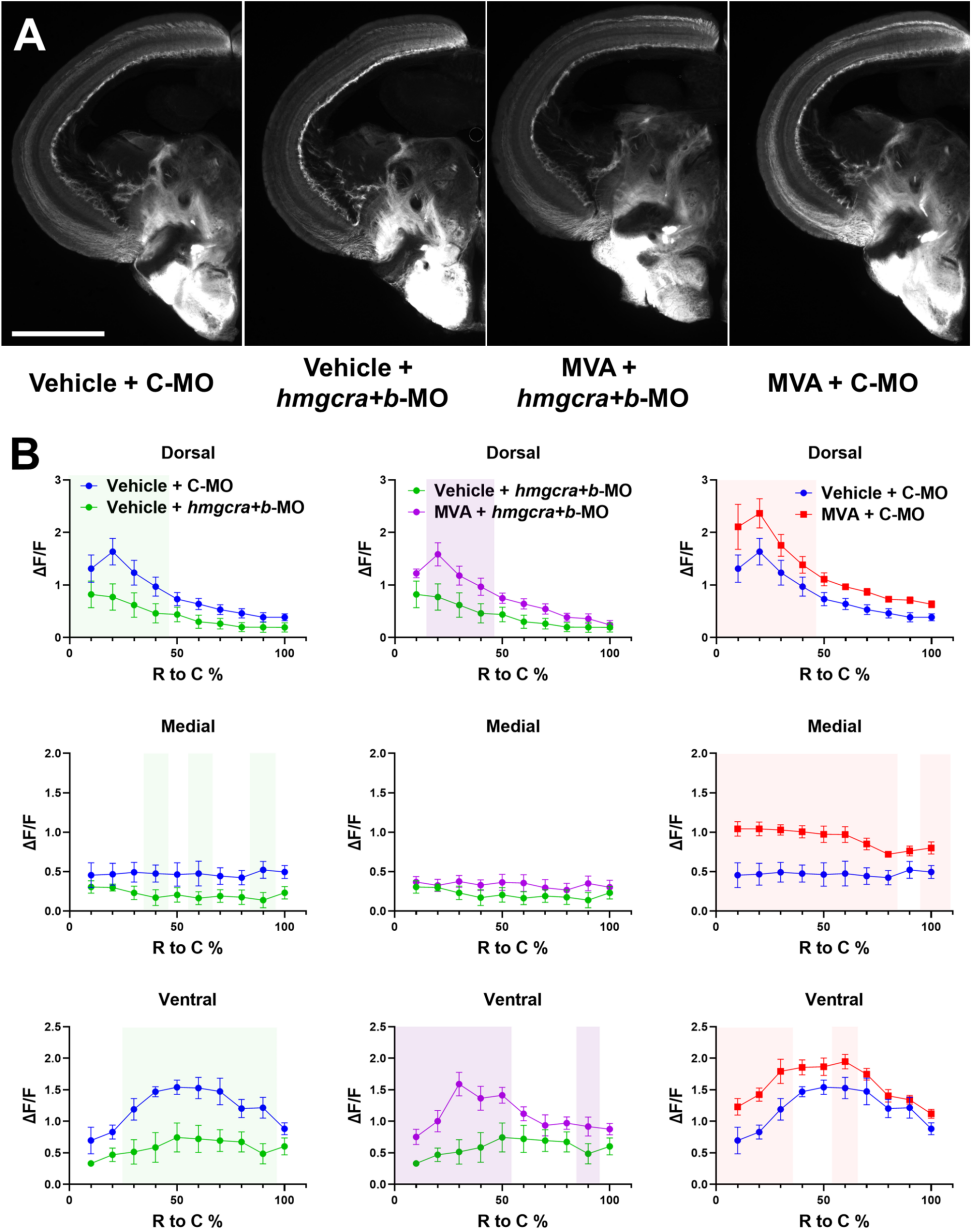
Axon regeneration into the optic tectum is dependent upon the mevalonate synthesis pathway. (**A**) Axon regeneration into the optic tectum is reduced upon *hmgcra* + *hmgcrb* knockdown (*hmgcra + b*-MO), rescued by daily supplementation with mevalonate (MVA), and accelerated by MVA treatment alone. (**B**) Quantification of axon regeneration in each treatment group. Colored boxes represent *p* < 0.05 in treated groups compared with the C-MO and/or Vehicle treated group in the corresponding column by two-way ANOVA with Fisher’s LSD post hoc test, *n* = 5–6 for each group. Scale bar = 500 μm

Next, we investigated whether mevalonate synthesis, the first enzymatic steps of cholesterol synthesis, affects axon regeneration following optic nerve injury. To inhibit this pathway, we used *hmgcra* and *hmgcrb* MOs or simvastatin. We found axon regeneration was significantly decreased by each loss-of-function treatment alone (Figure S15). To test whether the observed effects were due to blockage of the mevalonate pathway as predicted, we performed a rescue experiment. Combined knockdown of *hmgcra* and *hmgcrb* by MO resulted in a significant decrease in axon regeneration at 7 dpi (Fig. 8A, B). This decrease was rescued by daily intraocular injections of mevalonate, the direct downstream product of *hmgcra/b* activity (Fig. 8A, B). To our surprise, we found that mevalonate treatment alone accelerated axon regeneration in the optic tectum (Fig. 8A, B). This data suggests that mevalonate synthesis is likely downstream of *srebf2-*regulated axon regeneration and that metabolic flux through this pathway or substrate availability may be limiting for the rate of regeneration.

## Discussion

Using LCM-seq we generated a transcriptome-wide dataset of the GCL response to ON injury at 3 dpi. This is a time after the initial injury response when most if not all RGCs are extending regenerating axons through the ON. Since the zebrafish GCL is highly enriched for RGCs (∼ 90%), this data likely represents a significantly increased RGC gene coverage over previous whole retina sequencing and microarray projects [3–5, 7, 49]. We detected several thousand differentially expressed genes, both up- and down-regulated at this timepoint. GSEA analysis with the WikiPathways database identified several enriched up-regulated and down-regulated pathways. The most highly down-regulated pathways included metabolic pathways (“Electron Transport Chain”, “Oxidative phosphorylation”, “TCA Cycle”, and “Glycolysis and Glucneogenesis”), suggesting a switch from oxidative phosphorylation to glycolysis may be occurring. Although we did not examine this observation in this study, recent studies show that glycolysis supports axon regeneration in mammals with loss of Pten and SOCS3 [50, 51] meaning this may be an important direction for future study. Among the upregulated pathways, “Cholesterol Biosynthesis” is at the top followed by pathways involved in cellular injury response (“Apoptosis Modulation by HSP70”, “FAS pathway and Stress induction of HSP regulation”, and “TNF-alpha NF-kB Signaling Pathway”, and cytokine signaling) as well as cell cycle (“DNA Replication” and “G1 to S cell cycle control”). It is not surprising to see injury and stress response pathways active in the GCL at this timepoint after injury. Heat shock proteins and cytokines have been shown to support axon regeneration in the zebrafish model [52–54] and survival or regeneration in mammals [55–59]. The appearance of cell cycle pathways is interesting since it has been suggested that zebrafish RGCs do not proliferate following optic nerve injury [60]. There are three possibilities here. First, invading microglia or macrophages may be proliferating in the GCL. However, we do not see increased gene expression of markers for these cell types at 3 dpi making this possibility unlikely. A second possibility is that RGCs are actively suppressing the cell cycle and thus increasing expression of a subset of genes within this pathway. A third possibility is that “cell cycle” genes may be functioning outside their canonical cell division role to be active participants in cell autonomous axon regeneration. The specific genes involved in these pathways are yet to be rigorously examined in the context of ON regeneration. In this study, we decided to focus on the most highly enriched up-regulated pathway, chol biosynthesis, and its master regulator, *srebf2*.

We show that Srebf2-driven expression of genes involved in the chol synthesis pathway are necessary for efficient axon regeneration in adult zebrafish. Similar pathway upregulation has been described in previous studies of ON and brain regeneration in zebrafish and suggested to be evolutionarily conserved in pro-regenerative species [7, 61, 62]. However, this observation had not been experimentally examined. We used both systemic Srebf2 antagonist treatment and morpholino antisense treatments, specifically in injured RGCs, to block *srebf2* function. This significantly reduced the rate of axon regeneration and functional visual recovery without compromising RGC survival. This effect could be rescued by conditional overexpression of a constitutively active *srebf2* transgene, although transgene expression itself was not sufficient to accelerate regeneration or visual recovery. Detailed LCM-seq, qRT-PCR, and in situ hybridization analysis of the GCL after *srebf2* loss-of-function, gain-of-function, and transgenic rescue confirm that chol biosynthesis pathway genes are downstream of *srebf2* in RGCs. To begin identifying the downstream mediators of this effect, we inhibited the mevalonate synthesis pathway by Simvastatin treatment or *hmgcra/b* morpholino gene knockdown. This led to a significant decrease in axon regeneration that could be rescued by mevalonate supplementation. Surprisingly, intraocular mevalonate supplementation alone was sufficient to accelerate ON regeneration. These findings, in sum, demonstrate that Srebf2 regulation of the chol synthesis pathway in RGCs is pro-regenerative and that metabolite abundance is limiting to the rate of axon growth in vivo.

During neural development, Srebf2 and the chol biosynthetic pathway is relatively active in neurons but largely transitions to astrocytes and oligodendrocytes in the mature brain with low basal Srebf2 activity remaining in neurons [11, 63–65]. However, Srebf2 can be activated in neurons by insulin signaling or depletion of extra-neuronal cholesterol sources, demonstrating intact regulatory mechanisms and function in mature neurons [11, 64–66]. A recent publication even suggests that Srebf2 is at the top of an evolutionarily conserved transcription factor module in mature RGCs [67]. Our data shows that *srebf2* is upregulated in RGCs by 3 days post ON injury and stays elevated to 14 dpi. This correlates well with the known time course of ON regeneration in adult zebrafish [44]. It also suggests that Srebf2 activity is highest during the axon outgrowth phase of regeneration and lower, though still elevated, at the later synaptogenesis phase. In mammals, Srebf2 and the chol pathway have not been reported to be differentially expressed after ON injury in RGCs even after treatments that stimulate some axon regeneration [68, 69]. It is currently unclear if Srebf2 activation is part of the missing puzzle for successful axon regeneration in mammals or if there is an evolutionary divergence in the involvement of this gene in axon regeneration.

Zebrafish RGCs are largely refractory to cell death after ON injury, while the majority of mammalian RGCs die within 2 weeks [8, 60]. We did not notice a significant change in GCL cell number in our initial optic nerve crush or our Srebf2 loss-of-function experiments at 3, 7, or 21 dpi. This is surprising given previous reports that a percentage of RGCs die after optic nerve transection but not crush in zebrafish [8, 60]. It is possible the method of our MO treatment with a complete crush and partial transection of the nerve sheath is more like crush alone versus full transection. This idea is supported by the hierarchical clustering of samples in Figure S13 where wt 3 dpi crush samples are intermixed with control MO treated samples. Whatever the explanation we do not see an overt loss of cells suggesting that Srebf2 function is not critical for RGC resilience after axonal injury in zebrafish but is specific for axon growth.

Srebf2 coordinates expression of the chol synthesis pathway which is highly upregulated at 3 dpi in our GSEA results. Using in situ hybridization, we found the key genes we tested (*hmgcra*, *hmgcrb*, *sqlea*,* npc1*, and *ldlrb*) are upregulated at 3 dpi. Three of these (*hmgcra*, *sqlea*, and *ldlrb*) are decreased by *srebf2*-MO2 treatment, demonstrating that these genes are downstream of *srebf2* and supports our LCM-seq findings. To our surprise, *hmgcrb* doesn’t respond to *srebf2* morpholino treatment, suggesting it’s response to injury is Srebf2 independent. However, *hmgcrb* is upregulated in the uninjured heat shock-induced nuclear *srebf2* overexpression group by qRT-PCR on whole retina mRNA but not above the level of injury alone in the heat shock-induced nuclear *srebf2* GCL LCM-seq data. *Hmgcra* is upregulated by injury, downregulated by *srebf2* morpholino treatment, and upregulated by constitutively active Srebf2 over expression, showing the canonical response to Srebf2 activity in the GCL and presumably RGCs. This suggests an evolutionary divergence in the regulation of the *hmgcr* duplicate genes after the teleost specific genomic duplication event that may be cell type specific. Future examination of the *hmgcra* and *hmgcrb* regulatory loci may identify changes in SREs or other regulatory elements mediating the observed effects and Srebf2 independent modes of chol synthesis pathway regulation. The data presented here highlights the importance of the function of these two genes in the chol synthesis pathway during axon regeneration in zebrafish.

[62] To begin dissecting which enzymes and metabolites in the chol synthesis pathway are involved in ON regeneration, we tested the importance of the mevalonate pathway, the most upstream component of the chol synthesis pathway. Inhibition of axon regeneration following *hmgcra* and/or *hmgcrb* loss-of-function or simvastatin treatment recapitulates the findings observed in the *srebf2* loss-of-function experiments. Furthermore, we observed a recovery in axon regeneration rates by rescuing this effect with the downstream product mevalonate. This strongly supports our conclusion that Srebf2 mediates its effects on axon regeneration through the chol synthesis pathway. However, it is still unclear which products of the pathway are necessary for efficient axon regeneration in zebrafish.

In mammalian optic nerve regeneration, the products of the chol biosynthesis pathway have been described to both support axon regeneration and inhibit it [70]. Chol itself is a multifunctional molecule involved in membrane fluidity, lipid raft formation, receptor signaling, intracellular vesicle trafficking and function, and mitochondrial function among others. Most or all of these could impact the efficiency of axon growth and regeneration. A well-studied alternative branch of the synthesis pathway is protein prenylation. Protein prenylation covalently modifies proteins with farnesyl or geryanylgeryanyl groups allowing them to be localized to the plasma membrane [71]. In isolated neuronal cultures, chol synthesis is necessary for neurite formation and growth [15, 72]. Therefore, it was surprising when statin drugs were described to enhance axon outgrowth on inhibitory substrates [16, 18]. There have been two proposed cell autonomous mechanisms for this observation: (1) Chol is necessary for lipid raft formation and efficient activity of inhibitory receptors in the growth cone [19] or (2) Protein prenylation of cytoplasmic proteins such as RhoA is necessary for the signaling mediating growth cone collapse [18, 73]. Our experiments cannot distinguish between these two possibilities. We found that Simvastatin treatment or *hmgcra/b* knockdown decreased regeneration in the zebrafish. These somewhat contradictory findings in fish versus mammals may be explained by the lack of an inhibitory environment in fish allowing for rapid regeneration of most if not all axons. Or the balance of inhibitory receptors or internal signal transduction machinery at the growth cone in RGCs may be evolutionarily different. From a raw materials perspective it makes sense that chol and other derivatives of this synthesis pathway should be necessary for membrane expansion during axon outgrowth. We propose that the enhanced axon outgrowth seen in mammals with chol synthesis inhibition is limited to overcoming the inhibitory environment and does not drive robust long-distance regeneration due to reduced presence of downstream chol synthesis pathway products. In fish, upregulation of the chol synthesis pathway allows for rapid axon extension that is significantly attenuated upon pathway inhibition. However, we did still observe axon regeneration with pathway inhibition albeit much attenuated. This regeneration rate may be equivalent to the regeneration seen in the mammalian models and be dependent on non-cell-autonomous sources of chol pathways products. Further study and careful examination of axon extension rates will be needed to test this idea.

To our surprise, daily intraocular injection of mevalonate in control MO-treated zebrafish accelerated axon regeneration into the optic tectum compared to the vehicle injection group. This effect was not observed with heat shock-induced nuclear *srebf2* overexpression itself, suggesting the pathway metabolites are limiting to axon regrowth. This supports our hypothesis that in general the chol synthesis pathway is providing raw materials needed for axon regeneration in zebrafish.

We did not distinguish the role of chol itself, protein prenylation, or other downstream pathways such as coenzyme Q10 biosynthesis, N-glycosylation, or hemeA synthesis in this study. A previous study in goldfish optic nerve regeneration found that cholesterol trafficking is increased in the regenerating visual system and inhibition of the final step in chol synthesis inhibited axon outgrowth from retinal explants [21]. However, the same inhibitor did not delay regeneration in vivo [21]. This observation may be explained by the necessity of other non-chol products produced by the pathway or alternative external sources of chol and other metabolites from astrocytes, oligodendrocytes, microglia, or recycled axonal debris. We have not explored these possibilities in the zebrafish system, yet.

Although the data presented here strongly supports a critical role for Srebf2 mediated chol biosynthesis pathway activity in driving efficient ON regeneration in zebrafish, some limitations of this study should be noted. First, LCM-seq of the GCL captures more than just RGCs mRNA. We estimate ∼ 90% of the GCL in adult zebrafish is RGCs with the remaining cells likely displaced amacrine cells and microglia. Other contributions could be made by oligodendrocytes and endothelial cells from the retinal nerve fiber layer and the Muller glia processes in the GCL itself. We cannot rule out contributions from these cell types in the differentially expressed genes detected in our assay. However, all chol pathway genes and *srebf2* transcripts measured by in situ hybridization showed strong and specific upregulation in RGCs. One of the strengths of the LCM-seq method is the ability to sequence the samples at high read depth not possible with current single cell methods. However, the ability to distinguish RGC subtype or other cell type specific responses is limited. Second, our experimental measurements were mainly based upon mRNA levels via LCM-seq, qRT-PCR, and in situ hybridization. Antibody availability for zebrafish is limited for the genes and pathway addressed in this study preventing quantification of protein levels or distribution. We also did not directly assess the levels of chol synthesis pathway products in RGCs during axon regeneration. This data will be especially important while determining the role of specific products in the observations presented here. The lack of antibodies and product measurement means we could not definitively determine the efficiency of our morpholino based gene knockdowns or our fatostatin and simvastatin treatments either. To validate these results, we depended on successful genetic or chemical rescue experiments, but cannot be certain whether we had partial or complete inhibition of each gene or protein prior to rescue. A third consideration when interpreting the data presented here is whether the effects we observed were caused by RGC autonomous effects or systemic effects due to the method of experimental manipulation. This is especially important given the known role of cholesterol trafficking between cells in the CNS and its effect on inflammation after injury [73]. We attempted to couple our systemic fatostatin or simvastatin treatments with paired morpholino-based RGC specific gene knockdown experiments to suggest the observed effects were RGC autonomous. Similarly, the observed rescue and increase in axon regeneration with intraocular mevalonate supplementation may not be RGC autonomous although the gene knockdown of *hmgcra* and *hmgcrb* should have been limited to RGCs. Finally, we did not see differential expression outside of the GCL of any pathway genes examined by in situ hybridization suggesting the observed effects of our experimental manipulations were at the level of RGCs. However, we cannot definitively rule out cell nonautonomous effects in some of our experimental observations.

## Conclusions

Our study observed the upregulation of chol synthesis pathway in RGCs during ON regeneration in adult zebrafish. We demonstrate that Srebf2 plays an important role in successful axon regeneration and vision recovery by regulating the chol synthesis pathway. We then show that mevalonate synthesis through Hmgcra/b at the top of the chol synthesis pathway is necessary for axon regeneration. Our findings suggest that chol synthesis plays a crucial role in successful adult axon regeneration and that supplementation with metabolites of this pathway could potentially facilitate axon regeneration.

## Electronic supplementary material

Below is the link to the electronic supplementary material.

Supplementary Material 1

Supplementary Material 2

Supplementary Material 3

Supplementary Material 4

## Data Availability

The datasets generated and/or analyzed during the current study are available in the NCBI Sequence Read Archive (SRA) as BioProject numbers PRJNA1139127 and PRJNA1145997. Materials are available from the corresponding author upon reasonable request.

## Abbreviations

bHLH: Basic helix-loop-helix
Chol: Cholesterol
CNS: Central nervous system
DEGs: Differentially expressed genes
DLR: Dorsal light response
Dpi: Days post injury
GCL: Ganglion cell layer
GSEA: Gene sets enrichment analysis
KEGG: Kyoto Encyclopedia of Genes and Genomes
LCM: Laser capture microdissection
Mev/chol: Mevalonate/cholesterol
MO: Morpholino
PEN: Polyethylene-naphthalate
PFA: Paraformaldehyde
RGC: Retinal ganglion cell
SFGS: Stratum fibrosum et griseum superficiale
SO: Stratum opticum
SRE: Sterol regulatory element
TF: Transcriptional factor
