# Supplementary material for: Srebf2 mediates successful optic nerve axon regeneration via the mevalonate synthesis pathway
Source: Mol Neurodegener. 2025 Mar 5;20:28. doi: 10.1186/s13024-025-00807-2 (PMC11883989; doi:10.1186/s13024-025-00807-2)
Supplement: Supplementary file 1 — Supplementary Material 1 [file 13024_2025_807_MOESM1_ESM.docx]

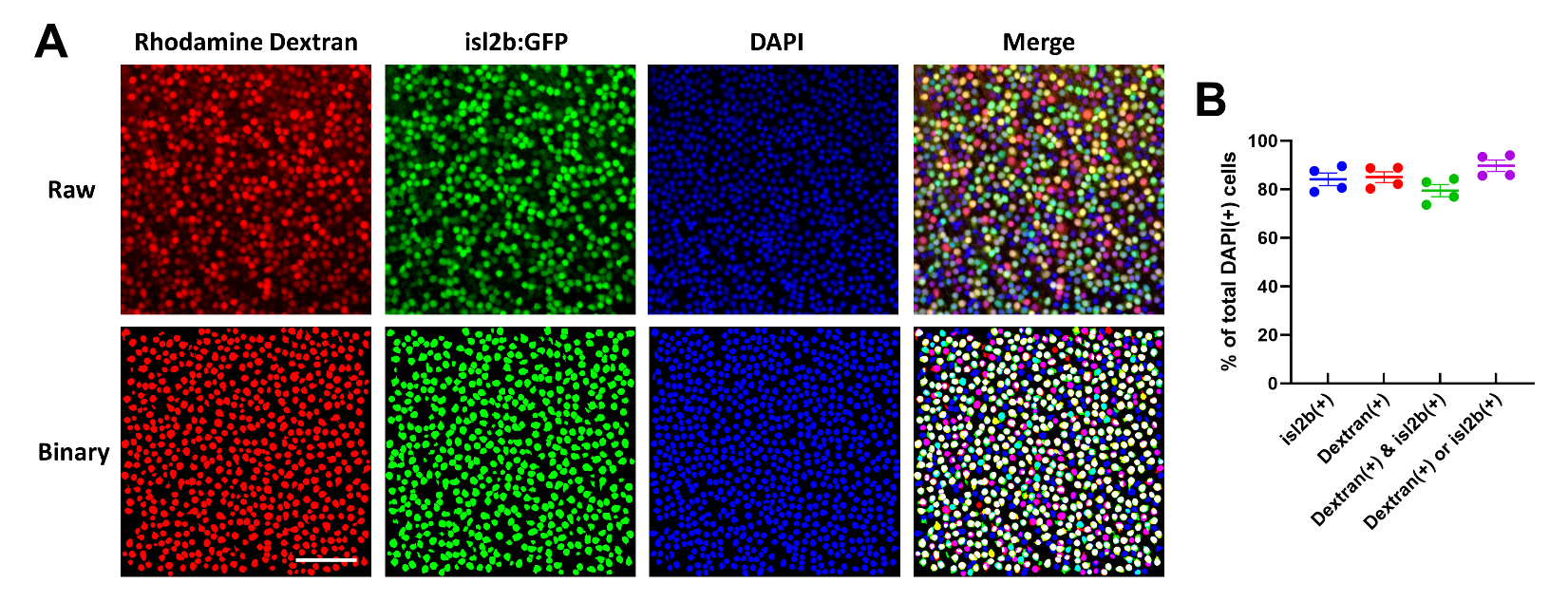


Figure S1. Retrograde labeling and *isl2b:GFP* transgene expression suggest the zebrafish GCL is made up of ~90% RGCs. A, Flat-mount confocal maximum intensity projections of retrograde tracing of the optic nerve into the retina with rhodamine dextran (3000 MW) in the *isl2b:GFP* line with DAPI counterstain used to label all nuclei in GCL. The upper panels show *isl2b:GFP* transgene expression with rhodamine dextran labeling in GCL. The lower panel shows binary cell outline images identified by ImageJ software to quantify labeled cells. B, Quantification of RGC percentages in the GCL, 84% of total GCL cells are *isl2b:GFP* positive, 85% of total GCL cells are rhodamine dextran positive, 79% of total GCL cells are co-labeled with dextran and isl2b:GFP, and 90% of total GCL cells are labeled by at least one RGC labeling approach. Four images from each retina were analyzed, one from each quadrant, n = 4 retinas, scale bar = 50 µm.


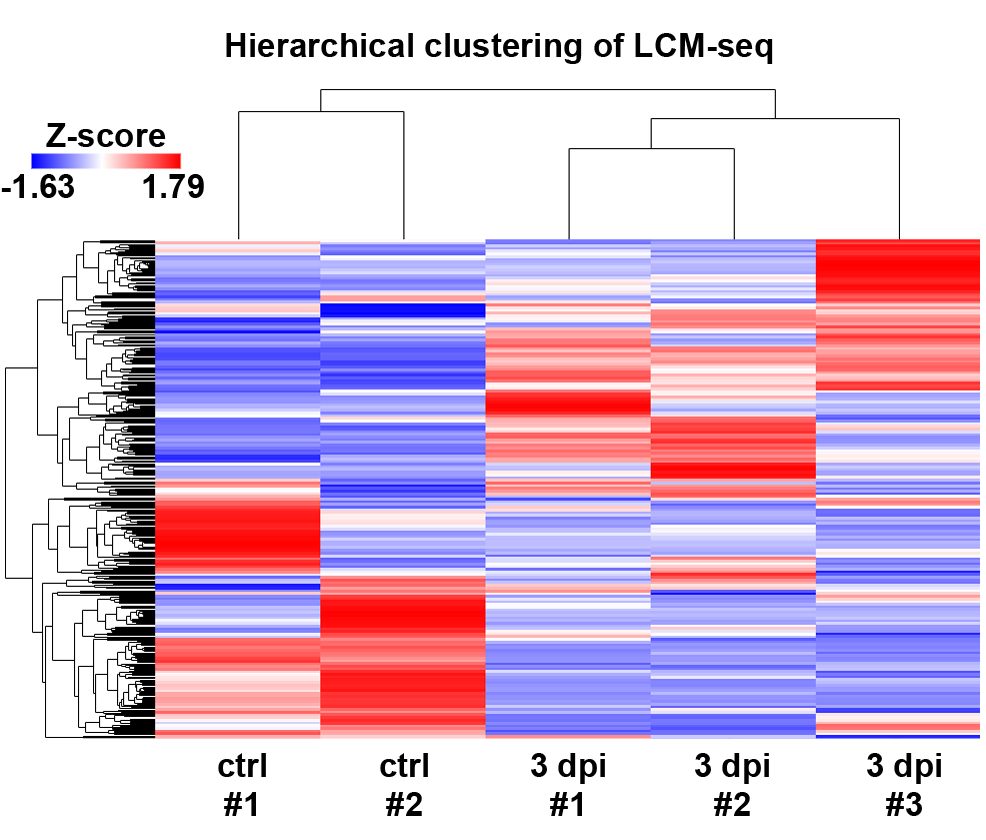


Figure S2. Hierarchical clustering of uninjured control and 3 day post injury (dpi) samples based on transcriptome-wide normalized gene expression. The two conditions clearly segregate into distinct groups. All samples are from male fish.


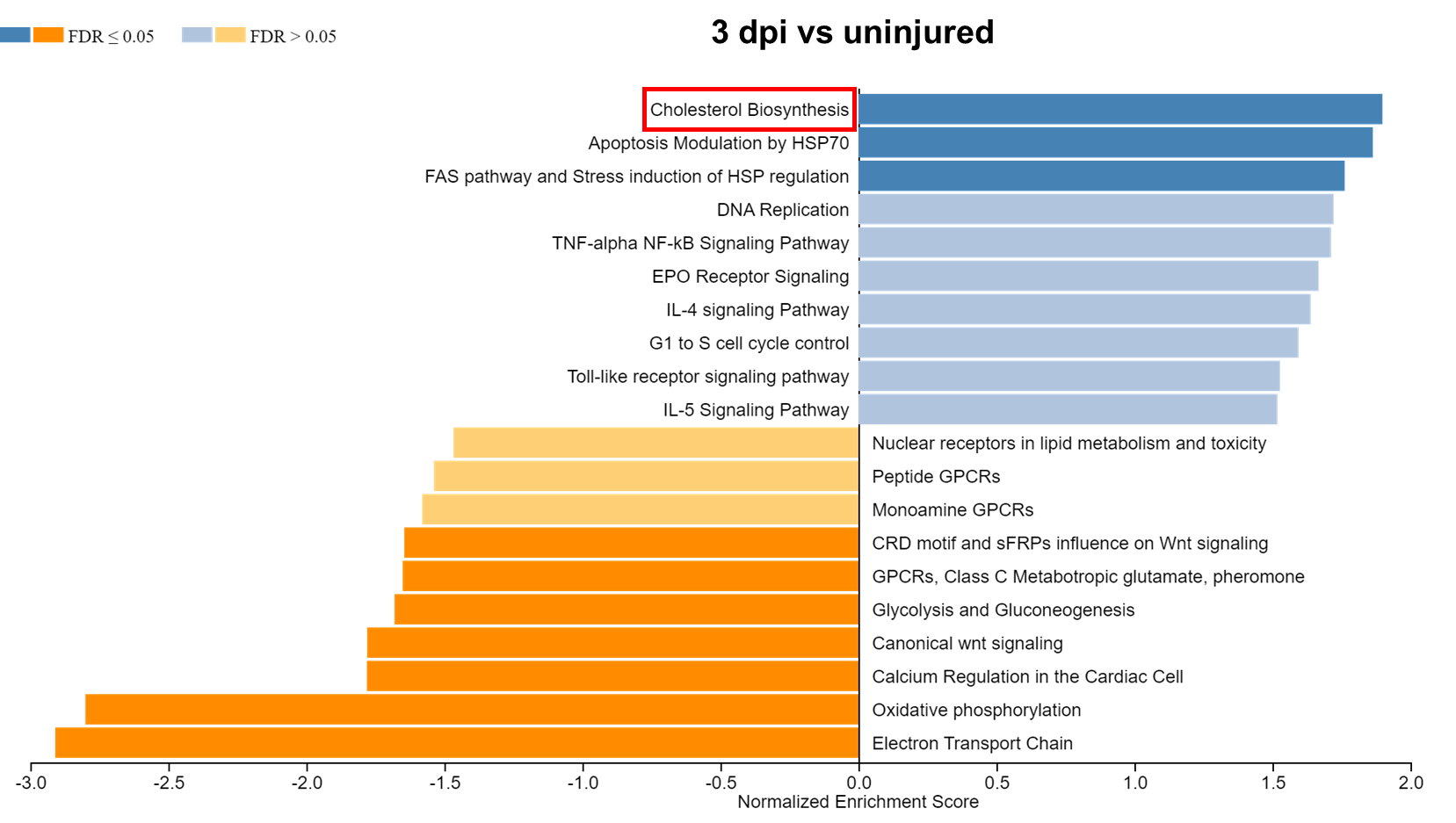
Figure S3. WikiPathways GSEA for 3 dpi vs. uninjured GCL.


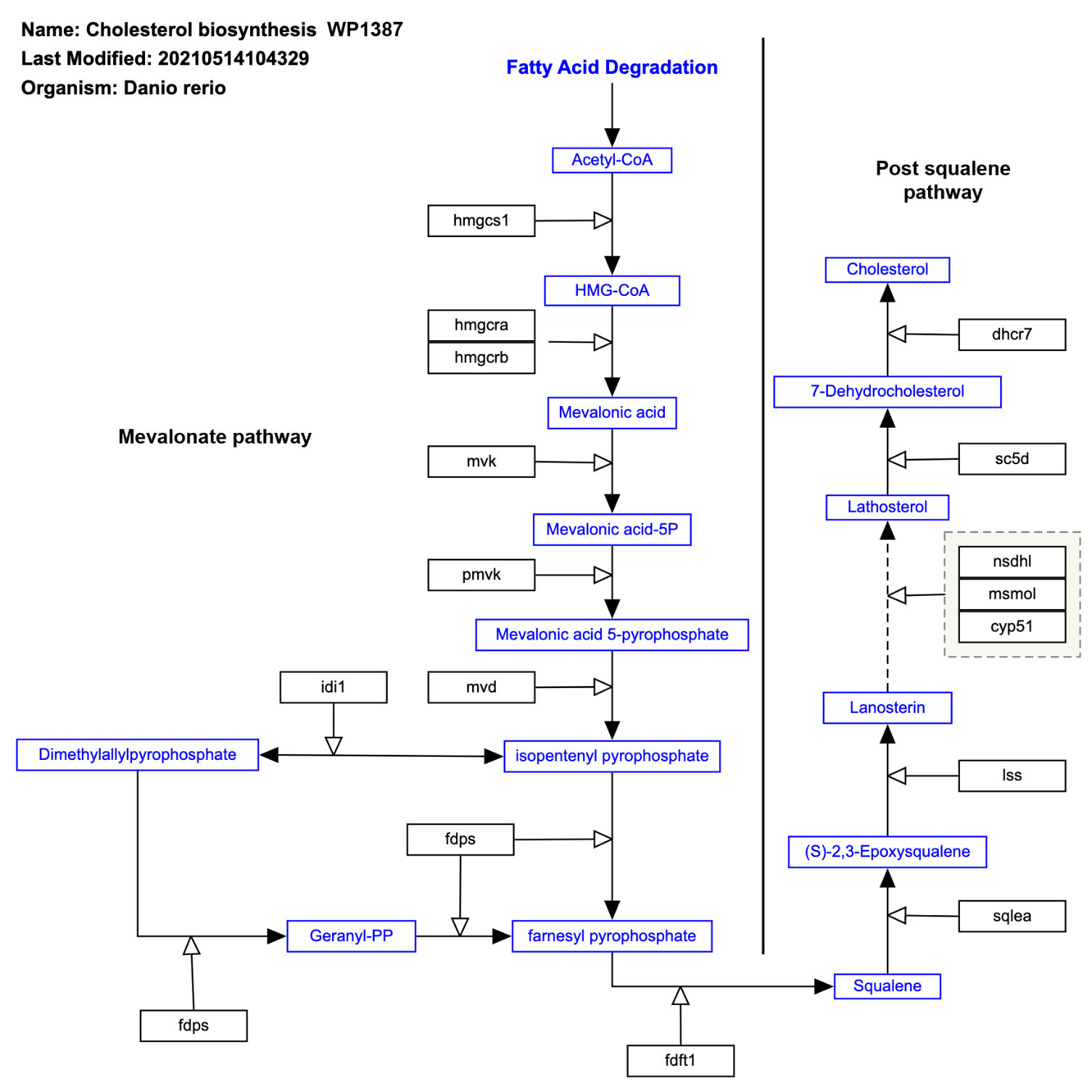


Figure S4. WikiPathways of Cholesterol biosynthesis with the Mevalonate pathway and Post squalene pathway highlighted.


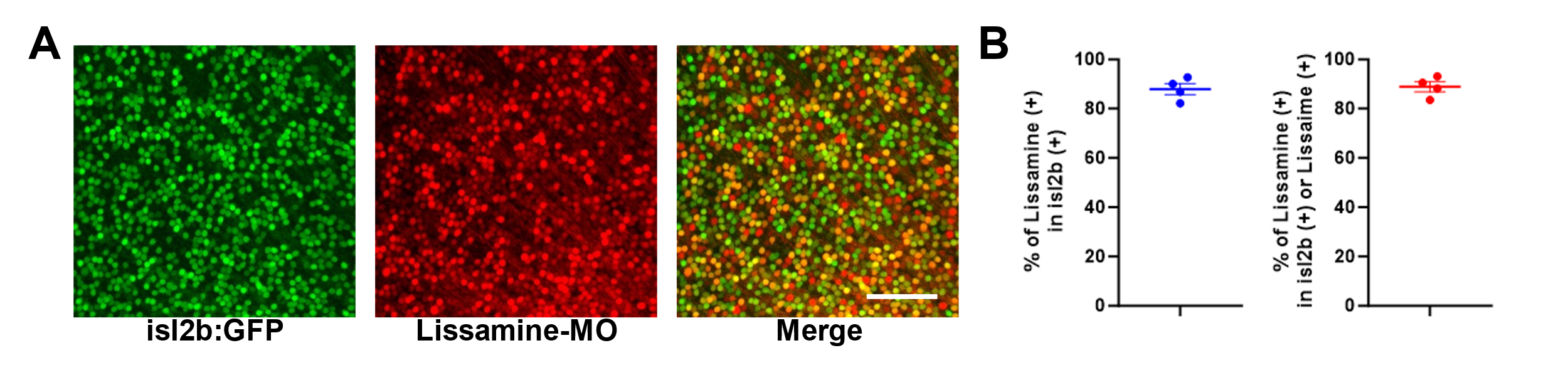
Figure S5. Morpholino delivery to the retina is 80-90%. A, representative images of lissamine-MO labeled RGCs in *Tg(-17.6isl2b:GFP)^zc7^* zebrafish retina. B, quantification of MO labeling efficiency in GFP positive RGCs or total RGCs with either GFP or lissamine positive cells.


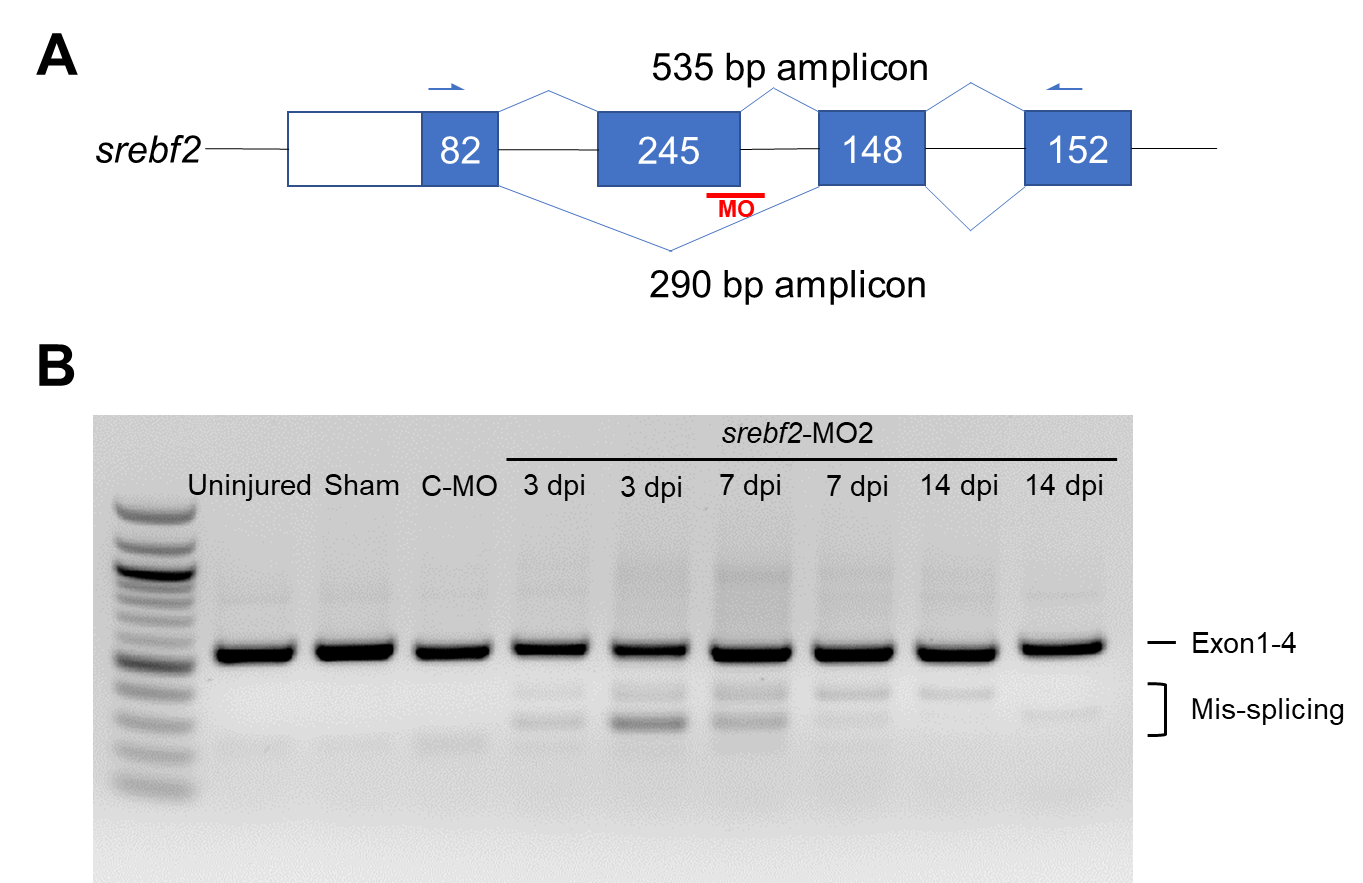
Figure S6. *srebf2* splicing MO (*srebf2*-MO2) validation. A, schematic of *srebf2*-MO2 target and primer target. B, RT-PCR gel of whole retina RNA at different days post RGC *srebf2*-MO2 delivery, ~300bp and ~400bp mis-splicing amplicons are observed from 3dpi to 14dpi *srebf2*-MO treated retinas in biological duplicates.


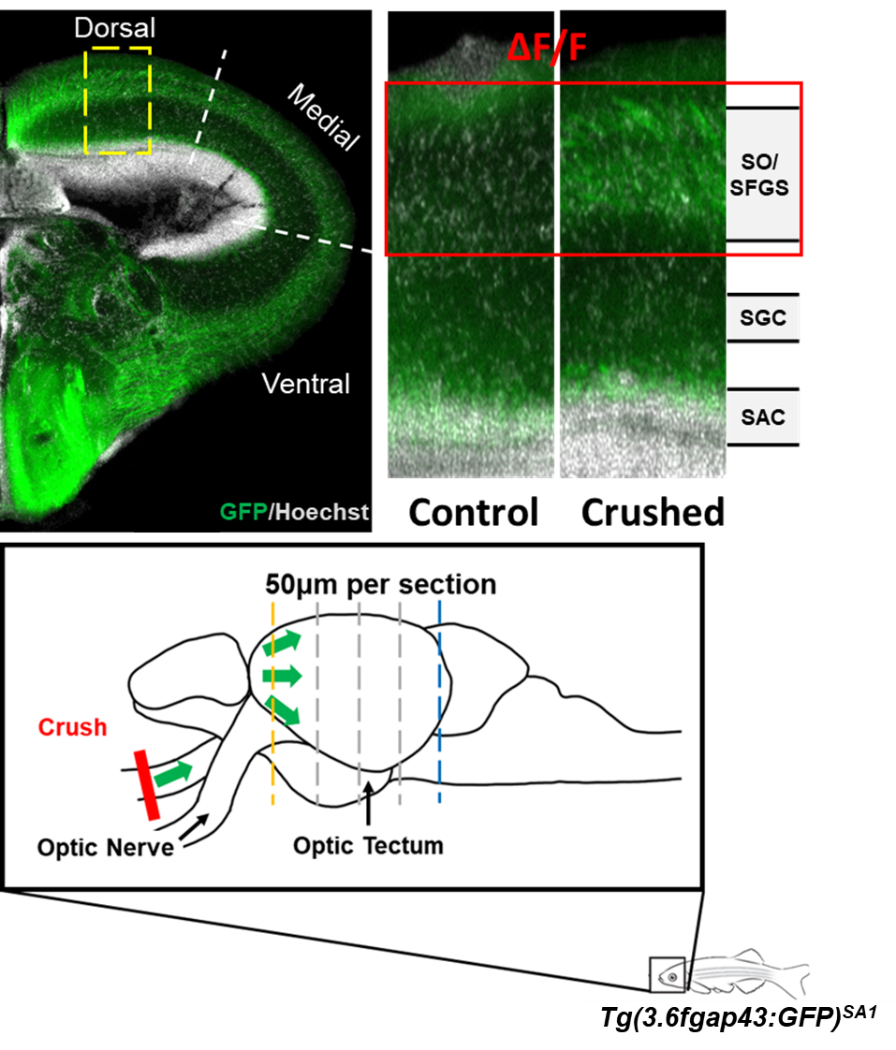
Figure S7. Method of quantification of optic tectum regeneration. We commonly measure all 50 μm sections harvested from each brain, approximately 15-20 vibratome sections. In the first phase of axon regeneration, the regenerating axon grows along the nerve sheath in the optic nerve; once the regenerating axon passes the optic chiasm, the axons split into two tracts; one reaches into the dorsal tectum, and the other becomes the ventrolateral optic nerve tract. Then axons from the two bundles spread and cover the medial region to finish the axon regeneration phase and move into the synapse refinement phase. This process recapitulates the RGC axon brain innervation during development. The ventrolateral optic nerve tract is perpendicular to the coronal section, making measurements of axon regenerating at the ventral tectum the most robust due to the optimal plane of section in this method.

Figure S8. *srebf2* loss-of-function inhibits ON axon regeneration at 3 days post injury in the ON. A, representative image of 3 dpi optic nerve post control morpholino (Ctrl MO) or *srebf2*-MO2 treatment. The white arrow indicates the injured site. The dotted line outlines both the regenerating optic nerve and the uninjured nerve to the optic chiasm. B, Quantification of axon regeneration in the ON with srebf2-MO1 treatment in the top graph, srebf2-MO2 in the middle graph, and fatostatin treatment presented in the bottom graph. * p < 0.05, ** p < 0.01, and *** P < 0.001 compared with control MO or vehicle at a certain distance to the injured site by two-way ANOVA with Bonferroni *post hoc* test. n = 5-7 for each group.
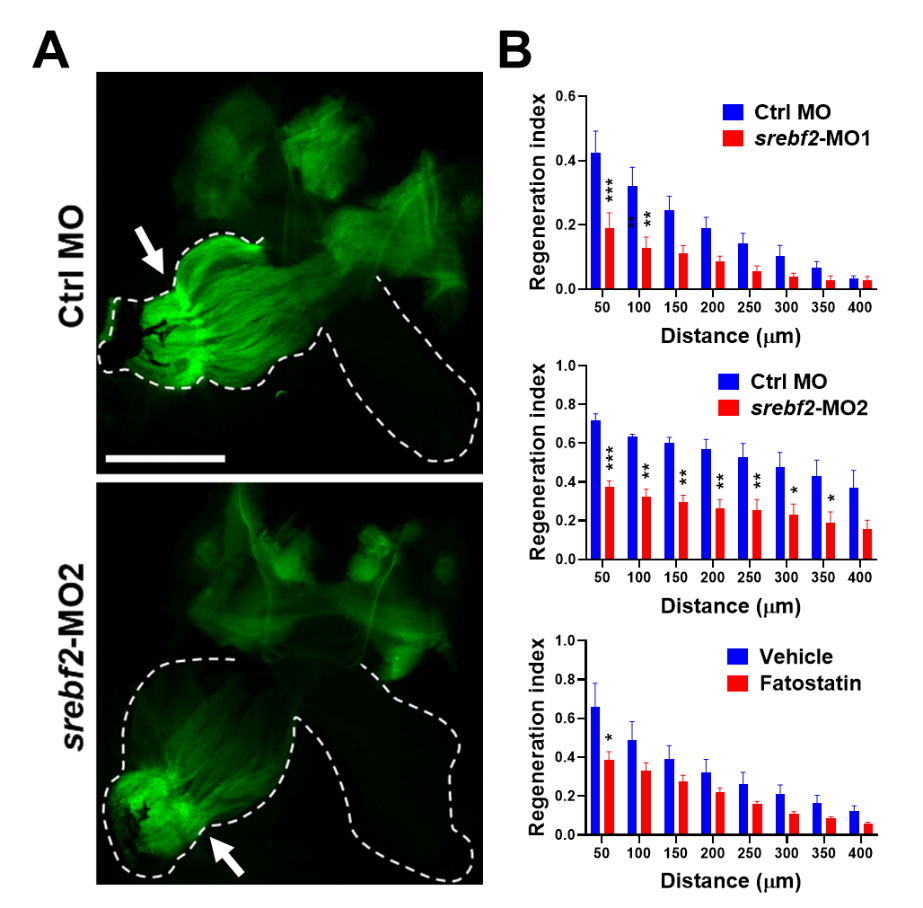


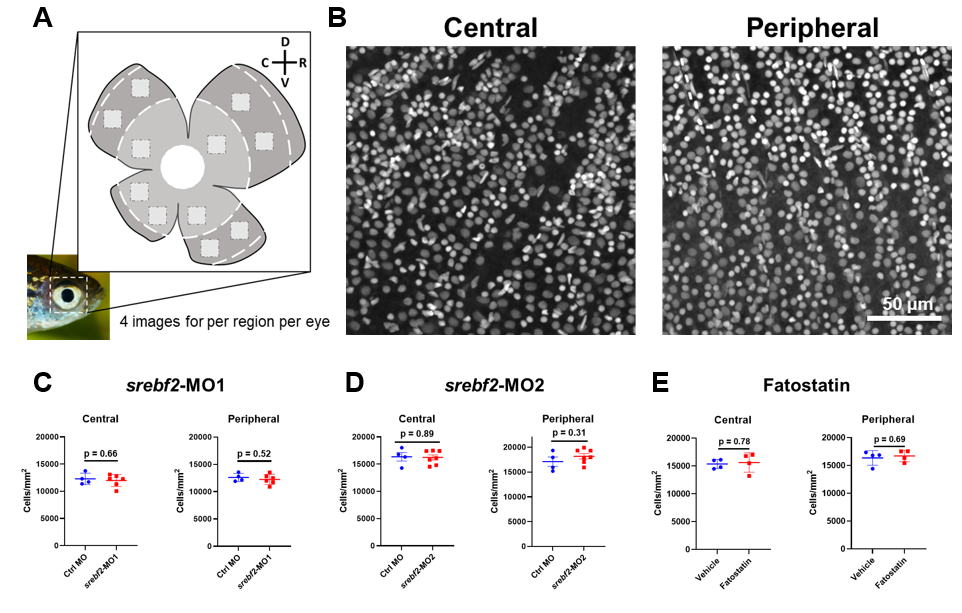


Figure S9. GCL cell count on *srebf2* loss-of-function retina. A, schematic of retina GCL image strategy. B, representative image of DAPI stained GCL image from the central and peripheral regions of retinal flat-mounts. By our estimation ~90% of cells in the GCL are RGCs (see Figure S1) C – E, cell count quantification of fatostatin and *srebf2*-MOs treatment retinas with t-test.


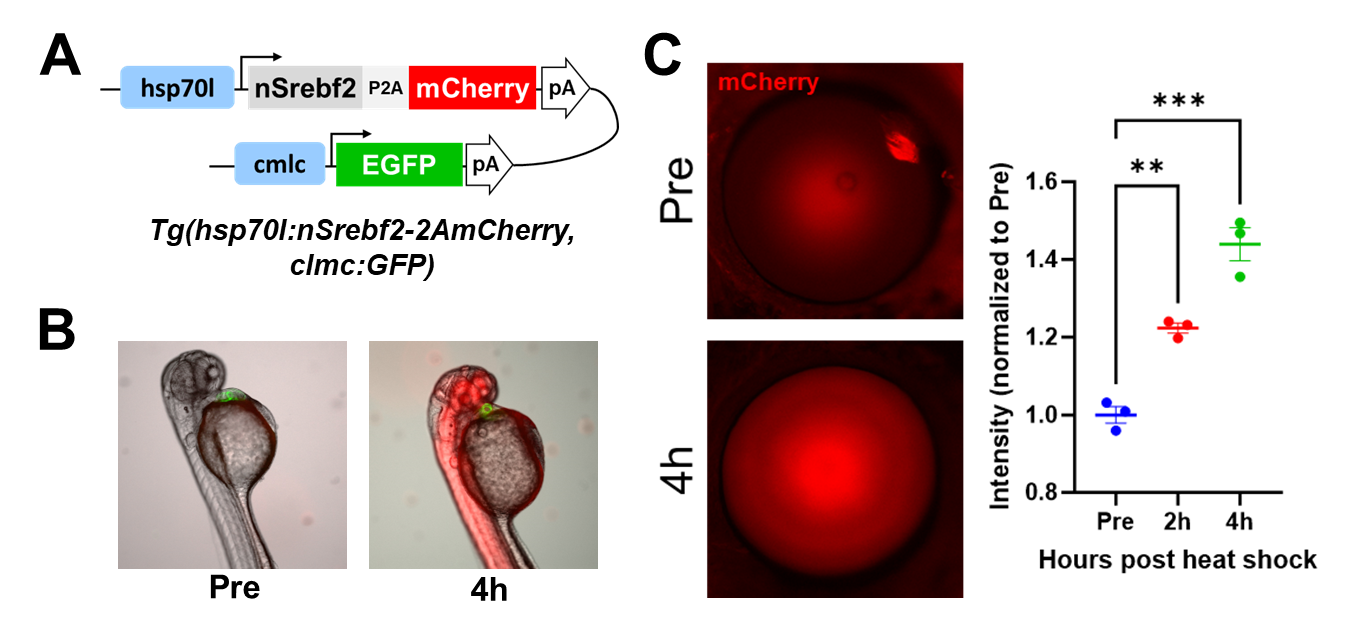
Figure S10. A, schematic of transgene design. B, mCherry expression is induced at 4 hours post heat shock in 4-day post fertilization embryos. C, mCherry expression in the lens & retina post heat shock in *Tg(hsp70l:nSrebf2-2AmCherry,cmlc:GFP)* adult zebrafish. ** p < 0.01, *** p < 0.001 by one-way ANOVA with Bonferroni *post hoc* test.


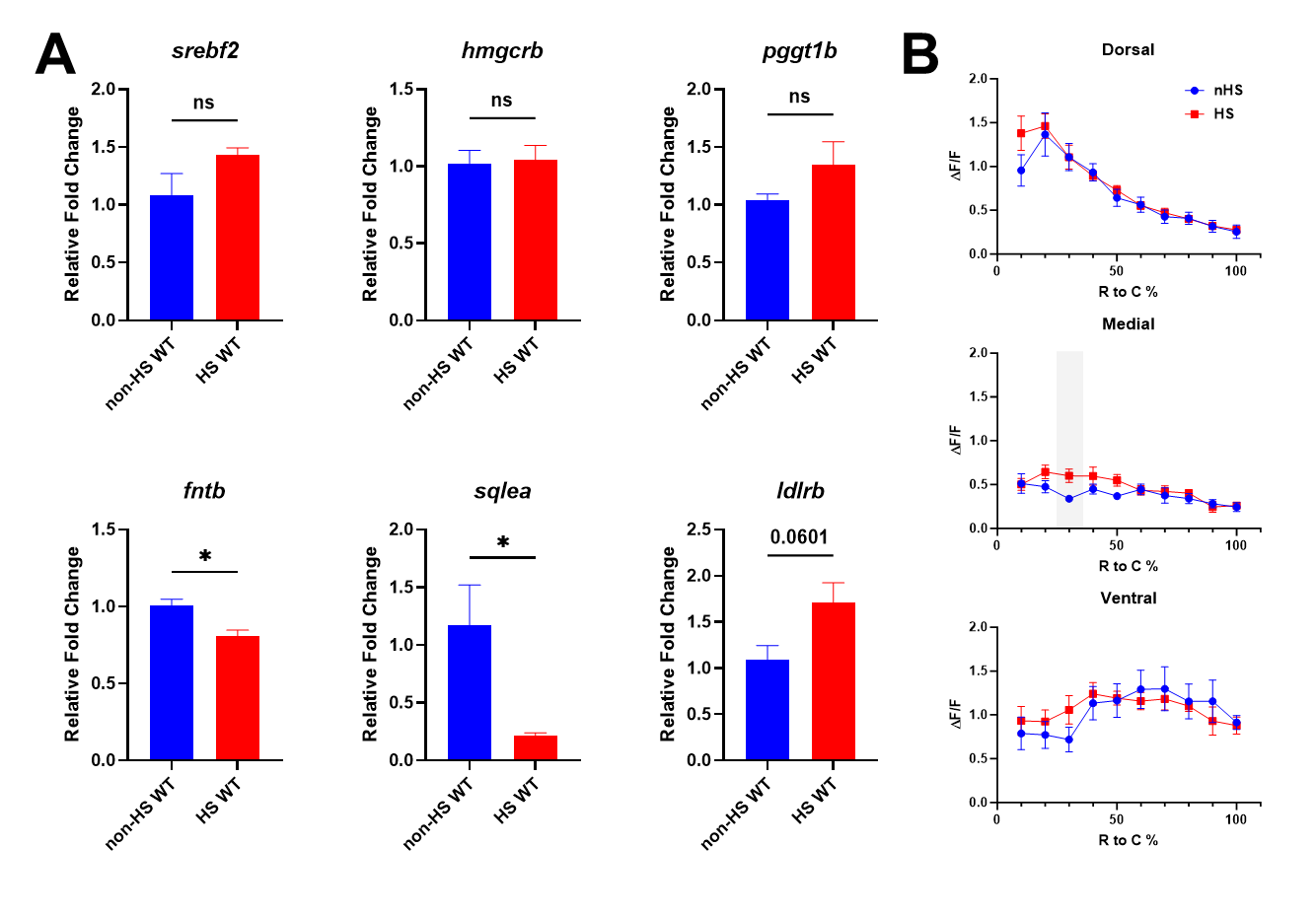
Figure S11. A, heat shock-induced mevalonate/cholesterol synthesis genes expression change on 4 hours post heat shock wild type (WT) zebrafish retina by t-test, n = 4 for each group. B, tectum regeneration on *Tg(3.6fgap43:GFP)^SA1^* zebrafish with heat shock procedure. A grey box indicates p < 0.05 at the corresponding rostral to the caudal region by two-way ANOVA with Fisher’s LSD *post hoc* test, n = 6 for each group, * p < 0.05.


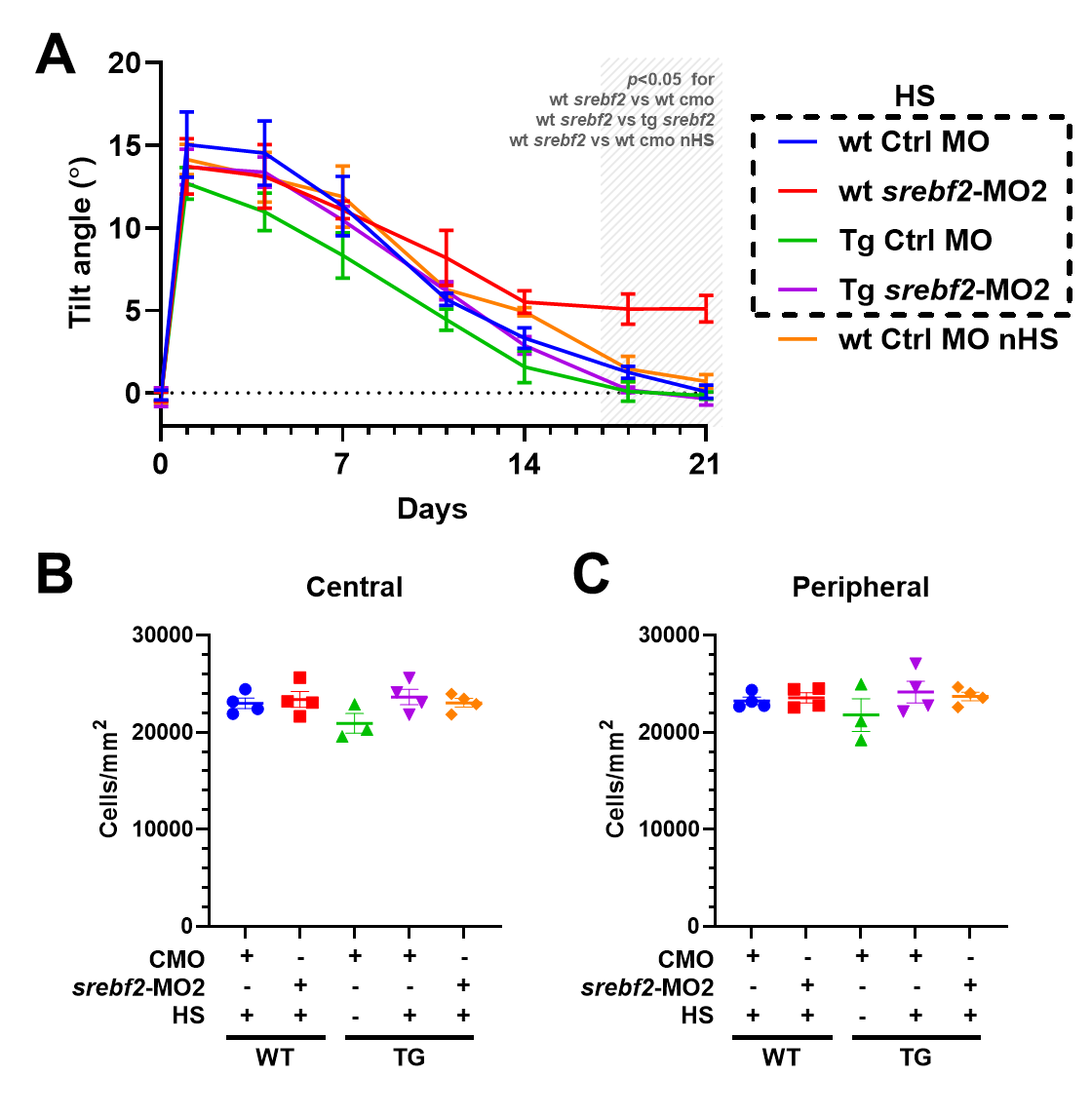
Figure S12. DLR & GCL cell count on *Tg(hsp70l:nSrebf2-2AmCherry;cmlc:GFP*) or wt zebrafish. A, DLR examines the effect of heat shock-induced nSrebf2 by two-way ANOVA with Bonferroni *post hoc* test, n = 5 to 6 for each group. The shaded area represents p < 0.05 for indicated comparisons. B - C, retina GCL cell count post DLR test by one-way ANOVA with Bonferroni *post hoc* test. HS indicates daily heat shock treatment; nHS indicates no heat shock treatment.


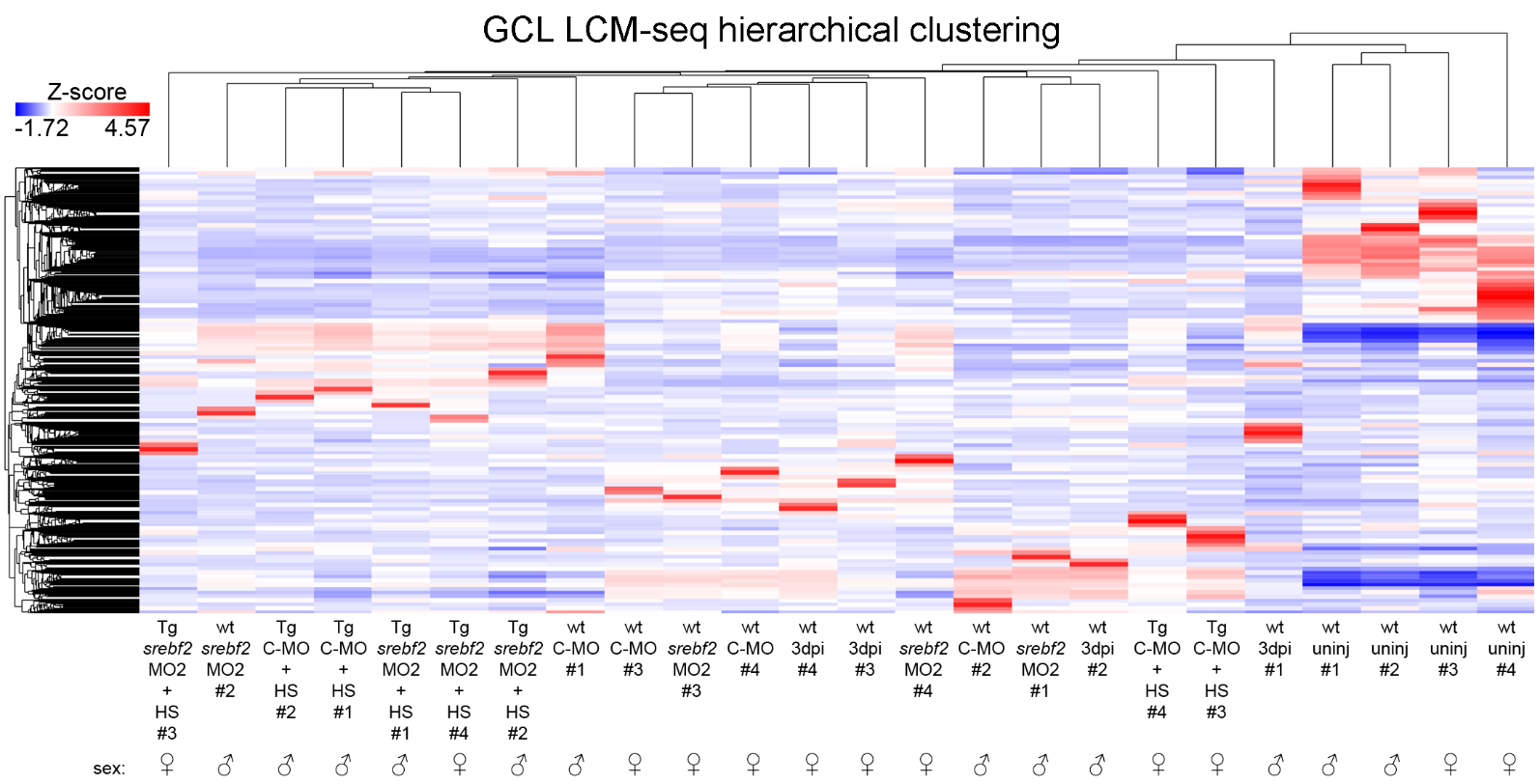


Figure S13. Hierarchical clustering of LCM-seq samples based upon treatment and normalized transcriptome-wide gene expression. Treatment groups include wild type uninjured (wt uninj), wild type 3 dpi (wt 3dpi), wild type treated with control morpholino (wt C-MO), wild type treated with *srebf2*-MO2 (wt *srebf2* MO2), *Tg(hsp70l:nSrebf2-2AmCherry;cmlc:GFP)* treated with control morpholino plus heat shock (Tg C-MO + HS), and *Tg(hsp70l:nSrebf2-2AmCherry;cmlc:GFP)* treated with srebf2 MO2 plus heat shock (Tg srebf2 MO2 + HS). N = 4 per treatment with 2 females and 2 males in each group. Samples are broadly grouped into three clusters based upon treatments (uninjured on the right side, optic nerve crush and cut/crush with morpholino treatment in the center, and heat shock treated samples on the left). This suggests the largest difference between groups is injured/regenerating versus uninjured. Secondarily, within the injured/regenerating groups, the robust heat shock response dominates clustering with two outlier samples having no heat shock response (Tg C-MO + HS #3 and #4) and two non-heat shock samples showing a heat shock like expression pattern (wt C-MO #1 and wt srebf2 MO2 #2). We did not exclude any samples based upon this observation. Sex dependent differences are evident within “response” clusters but not between them suggesting injury and heat shock responses are not sex specific but that the sexes can be transcriptionally distinguished within clusters.


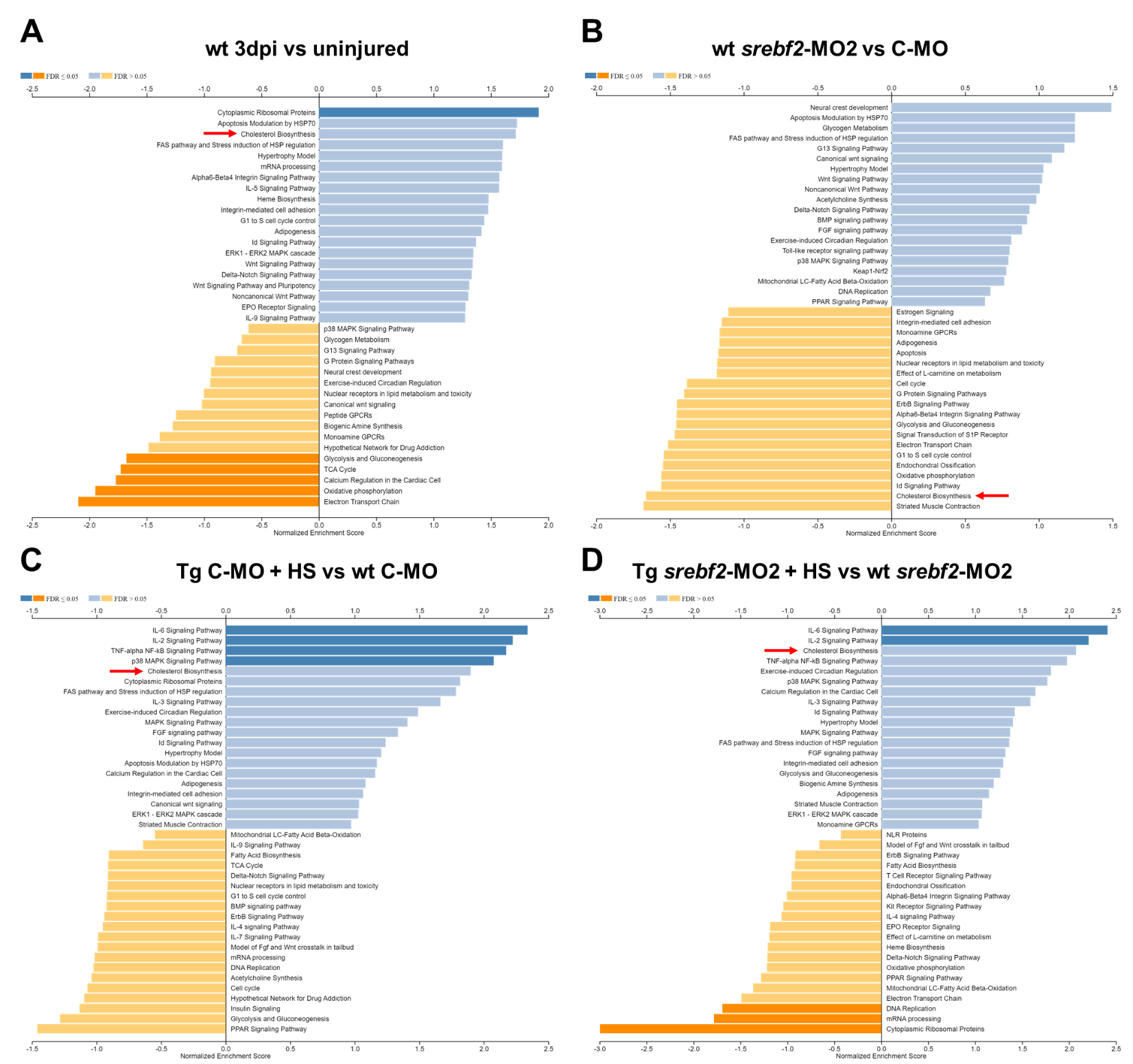
Figure S14. Complete WikiPathway GSEA for each comparison group. The red arrow indicates the cholesterol biosynthesis pathway.


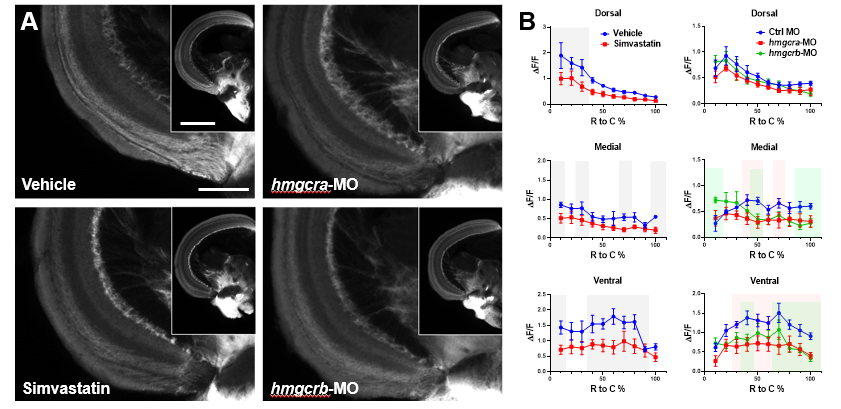
Figure S15. Optic tectum axon regeneration assay testing the mevalonate synthesis pathway. A, representative images of vehicle or simvastatin treated zebrafish optic tectum. B, optic tectum regeneration assay on simvastatin or *hmgcra* and *hmgcrb*-MO treated zebrafish. Colored boxes represent p < 0.05 in treated groups compared with the C-MO or vehicle group at the corresponding region by two-way ANOVA with Fisher’s LSD *post hoc* test. N = 5 - 6 for each group. Scale bar = 500 and 200 µm.
